# Genetic diversity and selection signatures of the beef ‘*Charolais de Cuba’* breed

**DOI:** 10.1038/s41598-018-29453-z

**Published:** 2018-07-20

**Authors:** Yoel Rodriguez-Valera, Gilles Renand, Michel Naves, Yidix Fonseca-Jiménez, Teresa Inés Moreno-Probance, Sebastian Ramos-Onsins, Dominique Rocha, Yuliaxis Ramayo-Caldas

**Affiliations:** 1 Facultad de Ciencias Agropecuarias, Universidad de Granma, Granma, Cuba; 2 GABI, INRA, AgroParisTech, Université Paris-Saclay, 78350 Jouy-en-Josas, France; 3 UR143, Unité de Recherches Zootechniques, Institut National de la Recherche Agronomique, Guadeloupe, France; 4 Empresa de Mejora Genética Manuel Fajardo, Jiguaní, Granma Cuba; 5 Animal Genomics Department, Centre for Research in Agricultural Genomics (CRAG), Campus UAB, Bellaterra, 08193 Spain; 6 Animal Breeding and Genetics Program, Institute for Research and Technology in Food and Agriculture (IRTA), Torre Marimon, Caldes de Montbui, 08140 Spain; 7 Departament de Ciència Animal i dels Aliments, Universitat Autònoma de Barcelona (UAB), 08193 Bellaterra, Spain

## Abstract

In this study, we used BovineSNP50 Genotyping BeadChip data to estimate the structure, putative ancestral origin as well as to identify regions with selective sweeps that may have had an important role in the adaptation to tropical conditions of the ‘Charolais de Cuba’ (CHCU) breed. According to a principal component analysis, CHCU samples cluster together with taurine breeds with an estimated 93% of *taurus* ancestral alleles. Despite the short period since importation, we detected differentiation (Fst = 0.049) between the French Charolaise (CHA) and CHCU. However, CHA breed was the closest breed to CHCU followed by other hybrids breed with a clear CHA origin. Linkage disequilibrium (*r*^2^) decay tends to be lower in CHCU compared to CHA probably due to a less intense artificial selection programs of CHCU. Signals of recent adaptation to tropical conditions between CHCU and CHA were identified. Genes mapping within those regions reflect different functions related to immunity, metabolic changes and heat tolerance (CHCU) and muscle development and meat quality (CHA) that may have had an important role in the phenotypic differentiation of these breeds. Further studies will expand our knowledge on the molecular basis of adaptation of cattle to tropical conditions and molecular process associated with meat quality traits.

## Introduction

Domestic cattle breeds (*Bos taurus* and *Bos indicus*) descend from the extinct wild ox or aurochs (*Bos primigenius*)^1^. Cattle domestication is estimated to have occurred in two separated locations: the Fertile Crescent between 6000 and 2500 BC (taurine cattle) and then in the Indus Valley about 2000 BC (zebu cattle)^2,3^. It is generally accepted that *Bos taurus*, spread into Europe via two routes: a Mediterranean route and a Danubian (or Continental) route^4^. Cattles were among the first old-world livestock species that were introduced in the Americas, as early as during the second Columbus trip in 1493^5^. The first settlement occurred in the Caribbean islands. Among them, Cuba is the larger island (110 000 km²), which harbors genetic resources that are not well studied. The subtropical climate of Cuba is characterized by high heat periods, low feed inputs, presence of parasites and tropical diseases that are not optimum for *Bos taurus* breed. The importation from France by Cuban farmers of Charolais cattle (CHA) occurred at the beginning of the twentieth century. More specifically, in 1900, “*Marqués de la Real Proclamacion de Cienfuegos*” was the first farmer importing CHA. Nevertheless, Ignacio Casas Saumells was documented as the most successful farmer in the first middle of the twentieth century, who imported CHA bulls to Caimanes (Santiago de Cuba province) and San José del Retiro farms (Granma province) from 1919 to 1938^6^. Interestingly, since the importation, these animals have been maintained as a “pure” breed without mating with other local breeds, mainly at the “Manuel Fajardo” genetic center located in Jiguani (Granma province)^6^.

Phenotypically, CHCU animals are white and unlike CHA animals, they are hairless. The production systems in France and Cuba differ largely. There is a single management system in Cuba with few large herds where breeding is 100% by artificial insemination (AI) and females are grazing all year long. During the drought period, they may be complemented with sugar cane residues. Comparatively, in France, there is a large variety of production systems in relation with geographical conditions. There are also great differences in the use of AI among herds, with only 14% of the females bred by AI on average^7^. During the winter period, from October-November to March-April, CHA cows are kept indoor and fed stored forages. Marked differences exist between CHCU and CHA cattle performances^6,7^. In Cuba the females are calving evenly during the four seasons, (20% in autumn to 30% in spring) while most of the females in France are calving in autumn and winter (30% and 39% respectively) and very few in summer (9%). The CHCU females are calving more early and have a longer productive life than CHA females: 11% of the calving females are 2 year old in Cuba, but only 2% in France and cows calving at 7 years or older are 38% and 28% respectively in Cuba and France. Growth capacity of CHCU is significantly lower than CHA with weights of 34 vs 46 kg at birth and 290 vs 493 kg for heifer’s weight at 18 months. CHCU progeny are also less efficient during fattening and produce carcasses with poorer muscle shape and higher fat content^8^.

The genetic structure and variability of the tropical adapted “*Charolais de Cuba*” (CHCU) and their relationships to other breeds is unknown. A pioneer report was done using blood groups^9^ suggesting a high variability of CHCU, similar allele composition with CHA as well as differentiation to other cattle breeds found in Cuba such as Holstein, Santa Gertrudis, Creole and Zebu^9^. The authors also reported the presence of a low frequency allele (U’_1_) found only in Zebu, suggesting a putative *Bos indicus* introgression in the formation of the CHCU breed. The main goal of this study was to use BovineSNP50 Genotyping BeadChip data to estimate the population structure, the putative ancestral origin of CHCU as well as to identify regions with selective sweeps that may have had an important role in the adaptation to tropical conditions, in comparison to CHA breed.

## Results and Discussion

### Charolais de Cuba genetic diversity pattern

**Figure 1:**
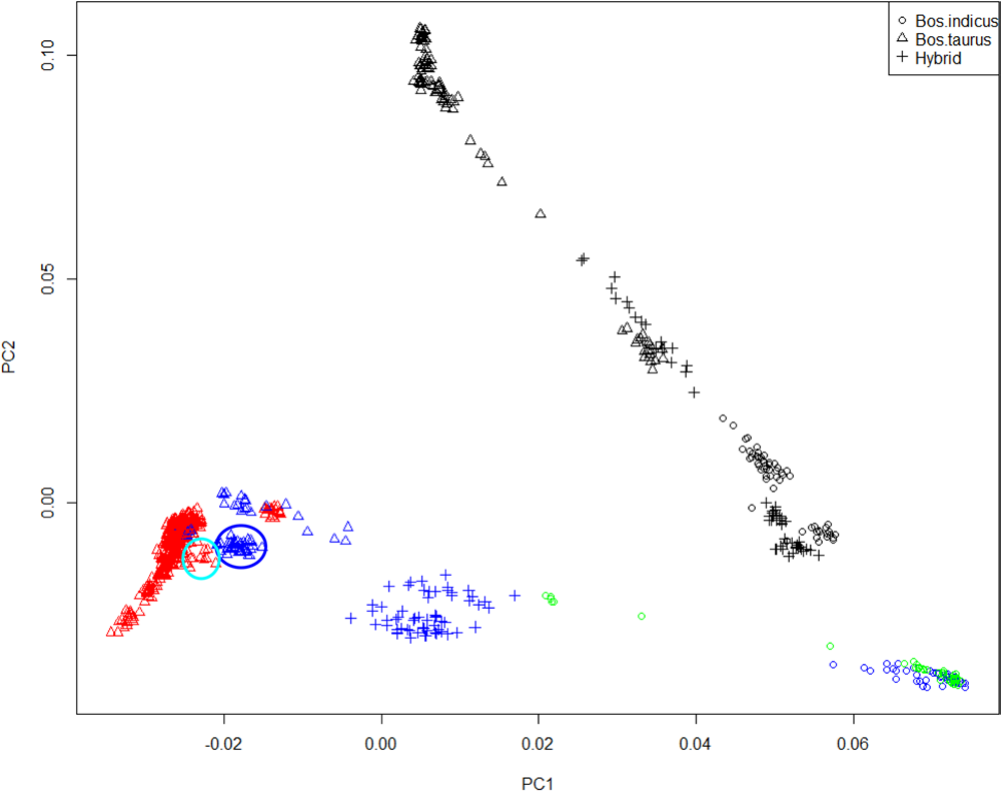
Principal component analysis using all samples. Individuals are grouped in *Bos taurus*, *Bos indicus* and Hybrid. Samples were colored according to geographic origin of breed; black: Africa, green: Asia, red: Europe and blue: America. CHCU and CHA samples are indicated using blue and light blue circle color respectively.

**Figure 2:**
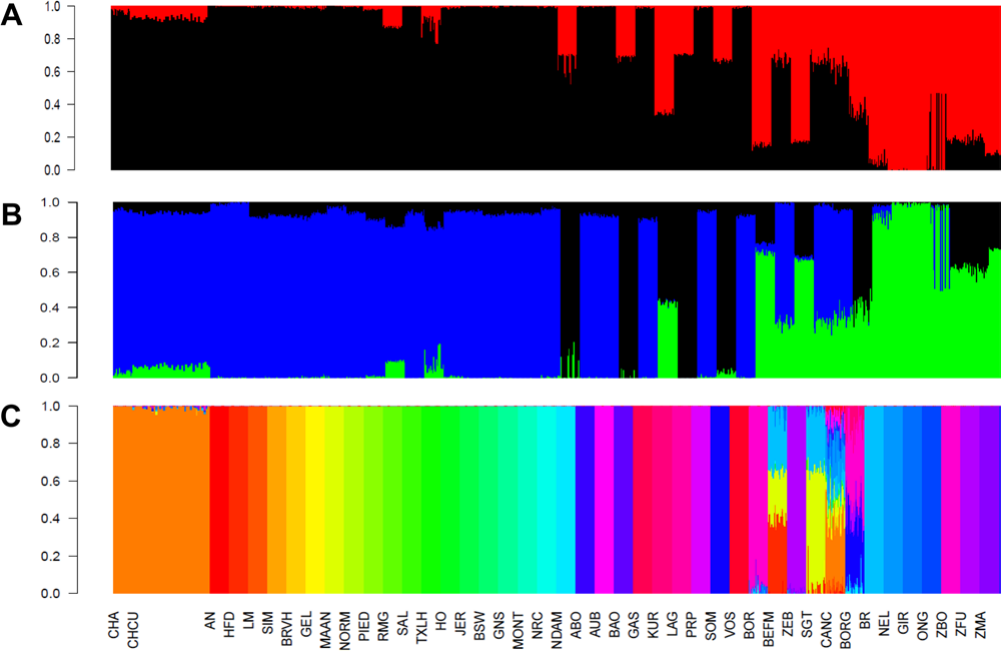
(**A**) Ancestry models with 2 ancestral populations (*K* = 2) the color correspond to: Black (*Bos taurus*) and red (*Bos indicus*). (**B**) 3 ancestral populations (*K* = 3) the color correspond to: blue (Eurasian *Bos taurus*), green (*Bos indicus*), black (African *Bos taurus*) and *K* = 37 (**C**). Breed names correspond to ABO: Abondance, AN: Angus, AUB: Aubrac, BALI: Bali, BAO: Baoule, BEFM: Beefmaster, BOR: Boran, BORG: Borgou, BR: Brahman, BRVH: Braunvieh, BSW: Brown Swiss, CANC: Canchim, CHA: French Charolais, CHCU: Cuban Charolais, GAS: Gascon, GEL: Gelbvieh, GIR: Gir, GNS: Guernsey, HFD: Hereford, HO: Holstein, JER: Jersey, KUR: Kuri, LAG: Lagune, LM: Limousin, MAAN: Maine-Anjou, MONT: Montbeliard, NDAM: N’Dama, NEL: Nelore, NORM: Normande, NRC: Norwegian Red, ONG: Ongole Grade, PIED: Piedmontese, PRP: French Red Pied Lowland, RMG: Romagnola, SAL: Salers, SGT: Santa Gertrudis, SIM: Simmental, SOM: Somba, TXLH: Texas Longhorn, VOS: Vosgienne, ZBO: Zebu Bororo, ZEB: East African Shorthorn Zebu, ZFU: Zebu Fulani, ZMA: Zebu from Madagascar.

A clear distinction between *Bos taurus* and *Bos indicus* samples was observed (Fig. 1), in agreement with^10–12^. According to PC1 and as expected, CHCU samples clustered within the *Bos taurus* group, close but differentiated to CHA (Fig. 1). In agreement with the PCA, a phylogenetic analysis supports the taurine origin of CHCU, suggesting also a close relationship with CHA (Supplementary Figure 1), is also interesting to note the close relationship between the African breeds (taurine and indicus) suggesting a clear differentiation between continents probably as consequence of the ascertainment bias. Afterward, ADMIXTURE was used to characterize the genetic structure of CHCU samples and to estimate their putative ancestral origin across *Bos taurus* and *Bos indicus* (Fig. 2). Regarding these two putative origins at *K* = 2, in agreement with the PCA, the estimated main ancestry of CHCU was *Bos taurus* (93%) with a small percentages (7%) of putative *Bos indicus* ancestry (Fig. 2A). Nevertheless, no homogenous ancestral composition in the CHCU samples was observed (*i*.*e*. two samples show 10% of putative *Bos indicus* alleles). Interestingly, our admixture analysis at *K* = 3 detects a putative introgression of African taurine alleles in CHA, and thus consequently in CHCU populations. This finding is in agreement with previously published results^10^. The unsupervised analysis considering the whole dataset proposed a *K* = 37 as an optimum partition number (Fig. 2C) suggesting a highly complex genetic structure or a high admixture level (for other values of *K* see Supplementary Figure 2). To be noted, the CHCU samples were assigned within the CHA cluster with an average putative CHA allele composition of 94% (according *K* = 37). In line with these observations^9^, using blood groups markers reported a similar allele composition between CHCU and CHA as well as a putative introgression of Zebu (*indicus*) alleles. We recognized the limitations of our study to trace *Bos indicus* origins (due the SNP ascertainment bias of the Illumina Bovine 50K BeadChip). However, based on the admixture analysis we cannot exclude a putative small introgression or contribution of *Bos indicus* in the establishment of CHCU breed as previously suggested^6,9^. Further analyses using a less biased source of information (*i*.*e*. whole-genome sequence data) will be needed to better estimate the putative *Bos indicus* introgression.

### Genetic differentiation among CHCU and others beef breeds

**Figure 3:**
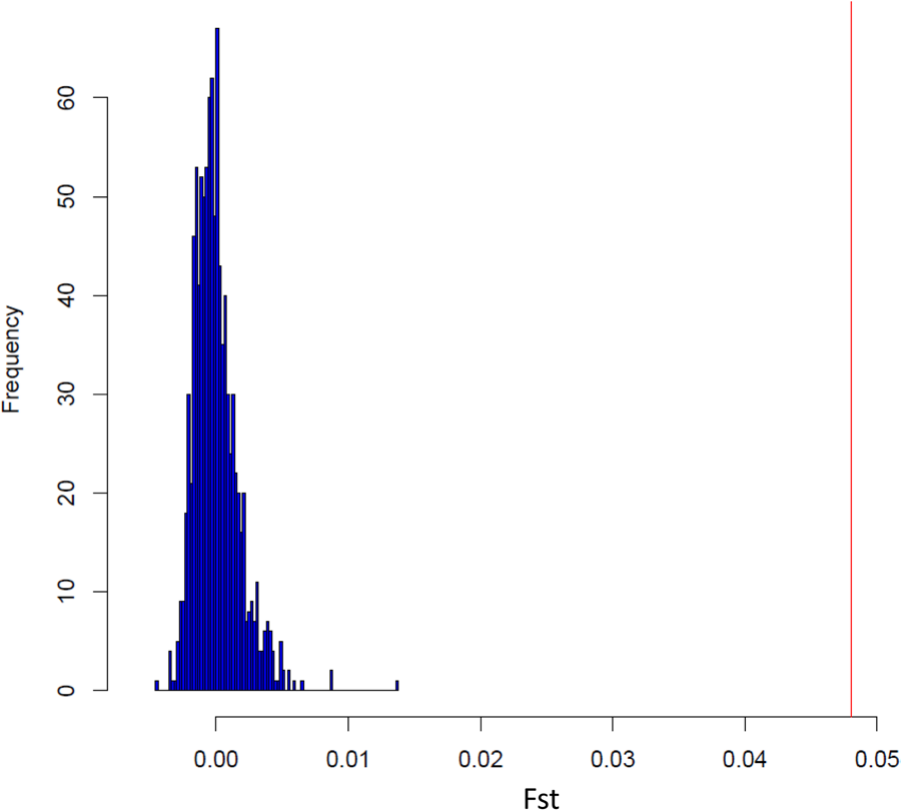
Distribution of the Fst after randomization of the dataset. The red vertical line represents the differentiation (Fst value) between CHCU and CHA.

To estimate the degree of differentiation between 19 beef breeds (180 *Bos taurus*, 120 Hybrids and 100 *Bos indicus* samples) the differentiation coefficient Fst^13^ was calculated. As expected the maximum Fst value was observed between *Bos indicus* and *Bos taurus* samples (Table 1). The Fst values were in agreement with those reported across other cattle populations^14^. Interestingly, despite the short period of time since the importation to Cuba of CHA (∼20 generations) differentiation between the CHCU and CHA was observed (Fst = 0.049). Actually, CHA breed was the closest breed to CHCU followed by Canchim (CANC, Fst = 0.05), Piedmontese (PIED, Fst = 0.06) and Limousine (LM, Fst = 0.07) breeds (Table 1). The significance of the observed differentiation (Fst = 0.049) between CHA and CHCU was validated by comparing the observed Fst value against the Fst distribution (1,000 replicates) after randomization of the dataset (Fig. 3).

**Table 1:** Matrix of Representation of the Fst corresponding to 19 beef breeds.

Breeds	BEFM	BOR	BORG	BR	CANC	CHA	CHCU	HFD	LM	MAAN	NEL	PIED	RMG	SGT	ZBO	ZEB	ZFU	ZMA
AN	0.1093	0.1693	0.1442	0.2035	0.0927	0.0924	0.1058	0.1336	0.1039	0.0932	0.2336	0.0897	0.1309	0.1022	0.1475	0.1717	0.1551	0.2024
BEFM		0.1049	0.1050	0.1115	0.0481	0.0762	0.0924	0.0794	0.0934	0.0823	0.1402	0.0805	0.1161	0.0543	0.0883	0.1168	0.0986	0.1482
BOR			0.0882	0.0960	0.0773	0.1319	0.1386	0.1767	0.1405	0.1512	0.1229	0.1280	0.1530	0.1045	0.0549	0.0839	0.0699	0.1091
BORG				0.1435	0.0769	0.1065	0.1157	0.1545	0.1090	0.1260	0.1722	0.0954	0.1235	0.1032	0.0310	0.0915	0.0377	0.1269
BR					0.0843	0.1662	0.1697	0.2110	0.1809	0.1845	0.0631	0.1674	0.1906	0.1120	0.0906	0.1268	0.1075	0.1536
CANC						0.0338	0.0529	0.1034	0.0557	0.0672	0.1056	0.0486	0.0866	0.0454	0.0590	0.0916	0.0712	0.1199
CHA							0.0497	0.1002	0.0522	0.0680	0.1970	0.0475	0.0960	0.0753	0.1084	0.1367	0.1168	0.1663
CHCU								0.1194	0.0656	0.0823	0.1942	0.0603	0.1042	0.0900	0.1171	0.1450	0.1245	0.1698
HFD									0.1125	0.1214	0.2410	0.0982	0.1424	0.1177	0.1559	0.1787	0.1637	0.2121
LM										0.0870	0.2095	0.0395	0.0909	0.0948	0.1156	0.1445	0.1229	0.1746
MAAN											0.2170	0.0721	0.1155	0.0592	0.1279	0.1567	0.1364	0.1853
NEL												0.1975	0.2202	0.1433	0.1166	0.1532	0.1328	0.1836
PIED													0.0722	0.0806	0.1028	0.1315	0.1110	0.1632
RMG														0.1161	0.1282	0.1573	0.1358	0.1905
SGT															0.0866	0.1177	0.0973	0.1454
ZBO																0.0727	0.0133	0.1021
ZEB																	0.0858	0.1293
ZFU																		0.1167

BEFM: Beefmaster, BOR: Boran, BORG: Borgou, BR: Brahman, CANC: Canchim, CHA: French Charolais, CHCU: Cuban Charolais, HFD: Hereford, LM: Limousin, MAAN: Maine-Anjou, NEL: Nelore, PIED: Piedmontese, RMG: Romagnola, SGT: Santa Gertrudis, ZBO: Zebu Bororo, ZEB: East African Shorthorn Zebu, ZFU: Zebu Fulani, ZMA: Zebu from Madagascar.

### Patterns of LD and detection of selective sweeps between CHA and CHCU breeds

**Figure 4:**
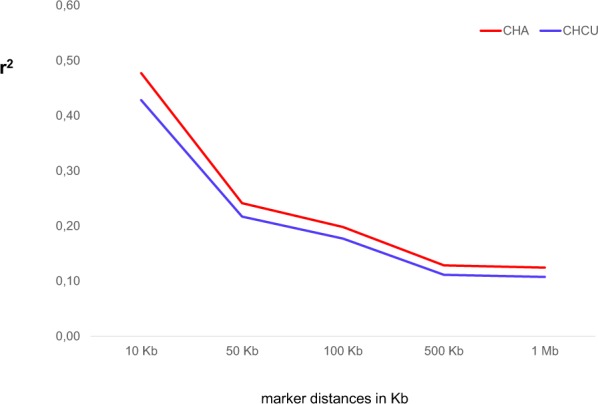
Linkage disequilibrium (*r*^2^) decay in CHA and CHCU populations.

In agreement with previous studies an inverse relationship between LD and genetic distance was observed^11,15^ with a rapid decline of *r*^2^ with genetic distance in both populations. The pattern of LD between CHA and CHCU was similar but at short distance the LD tends to be lower in CHCU compared to CHA (Fig. 4). For instance, at 10 kb the average LD (*r*^2^) for CHA and CHCU was 0.48 and 0.43, respectively. The lower LD observed in CHCU compared to CHA may suggest a stronger artificial selective pressure in CHA as a consequence of the breeding programs in France, compared to CHCU (less intense breeding program with artificial selection is established in Cuba). In agreement with the taurine ancestry of CHCU, the LD decay is equivalent to the values reported in taurine^11,15^ and higher to either composite or indicine cattle breeds^16,17^.

**Figure 5:**
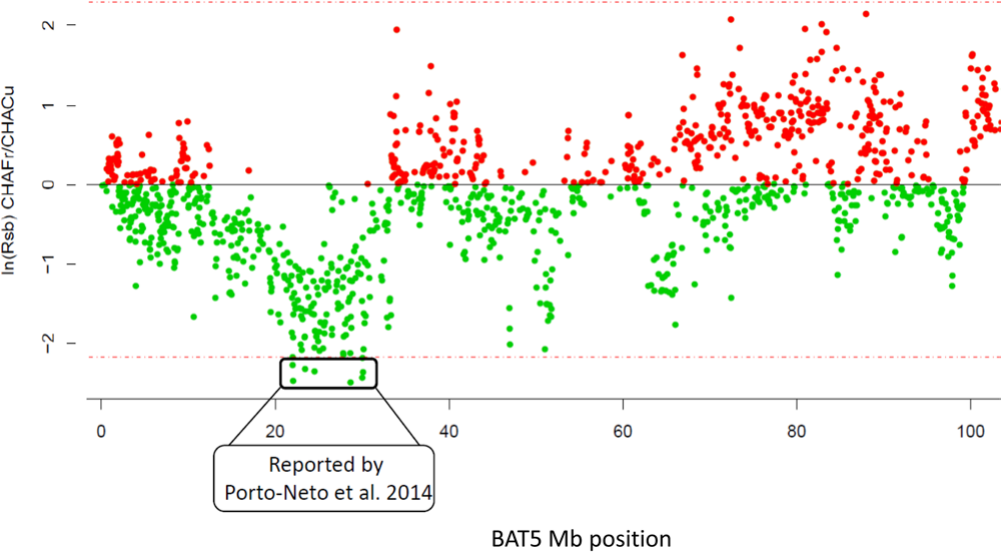
Graphical representation of the EHH pattern in the BTA5 chromosomal interval reported as associated with parasite resistance, yearling weight, body condition score, coat color and penile sheath score (Porto-Neto *et al*. 2014b). The x-axis represents the chromosomal position (Mb) and the y-axis the ratio of the ln(Rsb) CHA/CHCU.

To identify putative signals of adaptation to tropical conditions the Extended Haplotype Homozygosity (EHHS) was calculated and contrasted between the CHCU and the CHA. According to the chosen criterion (see methods), 409 SNPs corresponding to 104 different genomic intervals were identified in regions with extremely long haplotypes in relation to the expected pattern. 57% of those SNPs were intergenic variants, 32% were intronic variants, 9% were located upstream/downstream of genes and 1% were exonic synonymous variants (Supplementary Figure 3). A detailed examination of the literature reveals that ~55% of the intervals (57/104) overlap with selective sweeps and/or results of association studies related to adaptation to tropical conditions such as heat tolerance and parasite resistance (Supplementary Table 1). For instance, we identified two regions on BTA5 (intervals 20 and 21, Supplementary Table 1) overlapping with a chromosomal region previously reported as associated with parasite resistance, yearling weight, body condition score, coat color and penile sheath score^18^. As observed in Fig. 5 and Supplementary Table 2 extreme negative values of ln(Rsb) indicate slower EHH decay in CHCU breed than CHA and therefore suggest putative evidence of selection.

### Candidate regions and genes related to thermo-tolerance

Since its introduction to Cuba, CHCU animals have well adapted to the tropical conditions and have developed a tolerance to the high temperatures encountered on the island. It is noteworthy to point out that several of the regions selected in CHCU (intervals 38, 42, 48, 53, 87 and 97) are within or very closed to genomic regions associated to thermo-tolerance. For example, we identified 5 regions (intervals 38, 48, 53, 87 and 97) also found by Howard and collaborators^19^ while performing a genome-wide association study for body temperature, in US beef cattle. They did their study using 5-day period vaginal and tympanic temperature measurements in summer and in winter, on more than 200 crossbred heifers. Interestingly all the five regions shared between their and our studies are associated only to the summer period and we did not find any regions overlapping their regions found for the winter period. In addition, interval 42 overlaps with a region associated to sensitivity of milk production to environmental conditions^20^. Moreover, many genes located within the regions selected in CHCU have already been linked to heat stress. For instance, *AQP5*, located within interval 21, encodes a channel protein that selectively transports water through the plasma membrane of secretory and absorptive cells found for example in salivary or sweating glands^21^. Inactivation of *Aqp5* in mice has shown a key role for AQP5 in saliva fluid^22^. In addition, Sugimoto *et al*. have shown an upregulation of *aquaporin-5* expression in the salivary gland of heat-acclimated rats^23^. Cows can suppress the increase in body temperature dissipating heat by sweat evaporation but also by panting (salivation). *AQP5* is therefore a good candidate gene to explain the thermo-tolerance seen in CHCU. Another interesting gene is heat shock protein *HSPB1* (also known as *HSP25*, *HSP27* or *HSP28*) located within interval 95. *HSPB1* which encodes a small heat shock protein that functions as a molecular chaperone^24^, have been found in numerous studies among genes differentially expressed in heat-stressed animals from different species, including cattle^25^, zebu^26^ or buffalo^27^. It is worth noticing that this interval contains *ACHE*, another candidate gene potentially involved in thermo-tolerance. Indeed, knockout of *ACHE*, the gene encoding acetylcholinesterase, is responsible for hypothermia in mice^28^, while *ACHE* showed higher expression after exposing layer-type chickens to acute heat stress^29^. Interval 66 contains *UCP3* which encodes a mitochondrial uncoupling protein (UCP). UCPs are mitochondrial carrier proteins that catalyze a regulated proton leak across the inner mitochondrial membrane, diverting free energy from ATP synthesis by the mitochondrial F0F1-ATP synthase to the production of heat^30^. UCPs play therefore an important role in the regulation of cold acclimation, energy expenditure and diet-induced thermogenesis^31^. There were no significant differences in body temperature between wild type and *Ucp3* −/− mice, at thermo-neutral or cold (4 °C) conditions^32^. As these knockout animals were not exposed at high temperature, there is no evidence for a direct involvement of *UCP3* in heat acclimation. However, it has been shown that *Ucp3* expression was reduced in the hypothalamus of meat-type chicken after heat stress^33^ and that heat-stressed C2C12 murine myocytes displayed also a significant reduction of *Ucp3* mRNA expression compared to control^34^. *UCP3* therefore remains an interesting candidate gene to explain thermo-tolerance in CHCU, despite further work being needed. Overall, our results suggest that several of the regions selected in CHCU contain genes that might confer thermo-tolerance.

### Pathways related to adaptation to tropical conditions in CHCU and muscle development in CHA

**Figure 6:**
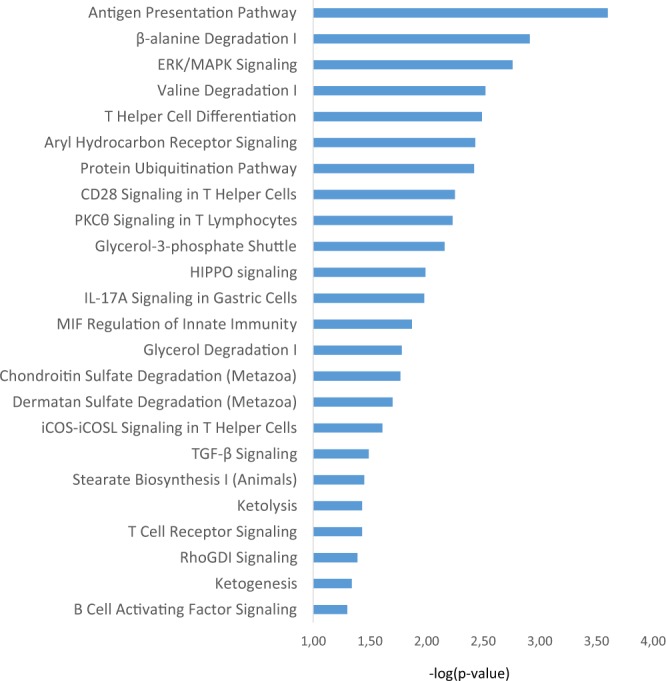
Over-represented canonical pathways considering all the EHH intervals.

153 genes were identified in the 104 genomic intervals (Supplementary Table 1). Pathways analysis reveal that those genes belong to functions related to immune system and other biological functions previously reported in selective sweeps studies^11,12,18,35–37^. The pathway analysis was done considering all the EHH intervals (Fig. 6), and considering the EHH signal in each breed separately (Supplementary Table 3). A detailed examination of the pathways identifies that the CHCU intervals show over-representation for immune related process. For instance, antigen presentation was the most significantly overrepresented pathway (Supplementary Table 3) which is involved in the processing of antigen, association of processed antigen with Major Histocompatibility Complex class (MHC) molecules and cell surface presentation. This process is central to the development of innate and adaptive immunity response. Interestingly, signature of selection in the MHC have been documented across several species^38–40^. Conversion of antigens from pathogens or transformed cells into MHC-MHC-II-bound peptides and I is critical for mounting protective T cell responses, and similar processing of self-proteins is necessary to establish and maintain tolerance^41^. Other pathways related to immunity such as T helper cell differentiation, B cell development, calcium-induced T lymphocyte apoptosis, role of NFAT in regulation of the immune response and B cell activating factor signaling were also over-represented. Based on the above observations it is tempting to speculate that these pathways have been essential for adaptation to tropical climate where the animals were exposed to tropical diseases, such as Anaplasmosis or Babesiosis, that have been reported during the introduction to *San Jose del Retiro*^6^. To be noted, the ERK/MAPK pathway and T cell apoptosis has been reported as mechanisms involved in the immune response against anaplasmosis^42^. Also T cell response and innate immunity are critical for the protection against babesiosis infection^43^. Metabolic pathways involved in ketolysis and ketogenesis were also overrepresented among the CHCU intervals. Ketolysis and ketogenesis pathways supply energy under circumstances such as fasting, in periods of negative energy balance^44^ which is often the case in tropical conditions having low quality and low availability of food. Interestingly, genes belonging to pathways related to immunity and metabolic processes (*HADHA*, *HADHB*, *HLA-DMA*, *HLA-DOA*, *HLA-DOB*, *PSMB8*, *PSMB9*, *TAP1*, *TAP*2, *APOB*, *GDF7*, *HS1BP3*, *LDAH*, *PUM2*, *RHOB*, *SDC1*) map within intervals with slower EHH decay in CHCU breed than CHA, suggesting evidence of selection (Supplementary Table 1).

On the other hand, in agreement with phenotypic differences with CHCU (in terms of muscle development and meat quality traits), as consequences of the intensive artificial selection through breeding program of CHA in France, over-representation of pathways relate to meat quality and muscle development such as β-alanine degradation, HIPPO signaling^45^, PPAR signaling^46,47^ were identified within the CHA intervals (Supplementary Table 3). Particularly among the CHA EHH intervals, β-alanine degradation was the most significant ones. Interestingly, β-alanine, as one of the precursor of carnosine, is associated with skeletal muscles development and plays important roles in cell metabolism^48,49^.

## Conclusions

We present here the first study describing the genetic diversity and structure of CHCU and their relationships to other breeds. CHCU samples reflect a clear *Bos taurus* origin and were mostly related to the CHA. Putative signals of recent selection between CHCU and CHA were identified and genes mapping within those regions reflect different selection emphasis/functions related to immunity, metabolic changes and heat tolerance (CHCU) and muscle development and meat quality (CHA) that may have had an important role in the process of adaptation to tropical Cuban conditions of CHA breed. Further studies on these pathways will expand our knowledge on the molecular basis of adaptation of cattle to tropical conditions and molecular processes associated with meat quality traits.

## Materials and Methods

### Samples and genotyping

A total of 40 CHCU were sampled by ear biopsy using a NextGen tissue sampling technology (Allflex). DNA of the 40 samples was extracted and genotyped using the Illumina Bovine 50K BeadChip (Illumina Inc., San Diego, USA) by LABOGENA (Jouy-en-Josas, France). Raw data were checked and analyzed with the GenomeStudio software (Illumina). Animal care and procedures were performed according European regulations about the protection of animals used in experimentation, following institutional guidelines for the Good Experimental Practices and approved by the Ethical Committee of the Institut National de la Recherche Agronomique (INRA).

### Structure of the CHCU population

To estimate the worldwide structure, diversity patterns and putative ancestral origin of the CHCU, we analyzed the public dataset corresponding to the study of (Decker *et al*. 2014), which provided genotype information of 43,043 autosomal single nucleotide polymorphisms (SNPs) scored in 1,543 animals distributed across 134 breeds. To avoid bias determined by unequal sample size, only those breeds having at least 20 samples were retained for further analysis (880 samples distributed across 43 breeds) (Table 2 and Supplementary Table 4). PLINK^50^ was used to merged the 40 CHCU samples with the 880 samples from^10^. The SNP positions were based on the UMD3.1 bovine genome assembly. After quality control, a total of 42,331 SNPs were used by excluding SNPs with minor allele frequency (MAF) <1% and missing genotypes >1%.

**Table 2:** Description of the samples used in the analysis.

Specie	Samples	Breeds
*Bos taurus*	620	30
Hybrids	120	6
*Bos indicus*	140	7
Total	880	43

### Analytical workflow

To visualize genetic distances between populations, principal component analyses (PCA) were performed with smartpca program from EIGENSOFT^51^. A phylogenetic analysis was done with MEGA7^52^ using the Neighbor-Joining method^53^. The robustness of the tree was supported by bootstrap (100 bootstraps) analysis. The evolutionary distances were computed using the p-distance method^54^ and are in the units of the number of base differences per site.

To examine potential origins of CHCU, the Maximum Likelihood approach implemented in ADMIXTURE^55^ was employed. Initially, ADMIXTURE 1.2 was run in an unsupervised manner with a variable number of clusters *K* = 2–44. Following the authors recommendations the lowest 10-fold cross-validation values were used to choose an optimum *K*-value. Additionally, a partial supervised approach was employed where assuming known ancestry *K* for samples corresponding to well-known established pure breeds. Before the PCA and ADMIXTURE analysis, linkage disequilibrium between SNPs was pruned using the option ‘–indep-pairwise’ in PLINK v1.07^50^. A total of 24,061 markers were selected from these analyses. Afterwards, a genetic differentiation analysis was done considering only beef breeds (400 indiviuals, 180 *Bos taurus*, 120 *Bos taurus/Bos indicus* crossbreds (hybrids) and 100 *Bos indicus*, Supplementary Table 4). The genetic differentiation between those beef breeds was assessed by Fst values for each pair of sub-populations using the method of Weir & Cockerham 1984^13^. The significance of the observed Fst between CHA and CHCU was validated by comparing the observed Fst value against the Fst distribution (1,000 replicates) after randomization of the dataset.

### Identification of genetic regions under selection

In order to detect selection signals across the genome, the Extended Haplotype Homozygosity (EHHS) was calculated and contrasted between the CHCU and CHA as described in^56^. Following the author recommendations^56^, first the integrated EHHS (iES) was estimated for each SNP and independently per population. Then, the EHHS decay of a single site between two populations was compared by calculating the log ratio of iES in both populations (ln(Rsb)). The cut-off for selecting the significant SNPs was established by picking out those SNPs that represented the top 1% of the tail area of the empirical distribution. Then, the genomic intervals were defined considering only those regions with at least two consecutive SNPs with less than 1 Mb distance. Finally, the extent of linkage disequilibrium (LD) between each pair of SNPs, measured as *r*^2^, which is less susceptible to bias due to differences in allelic frequency was calculated using PLINK v1.07^50^. In both cases the whole set of 42,331 SNPs were employed without excluding markers (*i*.*e*. pruned by linkage disequilibrium between SNPs).

### Pathways enrichment analysis

In order to describe the most over-represented pathways and biological processes affected by putative selective sweeps, the genes mapping within putative selective sweeps were used to perform a pathway enrichment analysis using the Ingenuity Pathway Analysis (http://www.ingenuity.com/products/ipa) tool. The list of protein-coding genes was uploaded into the application. Then, each gene identifier was mapped to its corresponding gene object in the Ingenuity Pathways Knowledge Base (IPKB). Fischer’s exact test was employed to calculate a *p*-value, which determines the probability that each biological functions and/or canonical pathway is due to chance alone. Only the enriched pathways with adjusted *p*-values < 0.05 after Benjamini & Hochberg^57^ multiple testing correction were retained.

## Electronic supplementary material

Supplementary Information

Supplementary table 3
