# Supplementary material for: Genetic diversity and selection signatures of the beef ‘Charolais de Cuba’ breed
Source: Sci Rep. 2018 Jul 20;8:11005. doi: 10.1038/s41598-018-29453-z (PMC6054659; doi:10.1038/s41598-018-29453-z)
Supplement: Supplementary file 1 — Supplementary Information [file 41598_2018_29453_MOESM1_ESM.pdf]

Genetic diversity and selection signatures of the beef '*Charolais de Cuba*' breed

Yoel Rodriguez-Valera[1], Gilles Renand[2], Michel Naves[3], Yidix Fonseca-Jiménez[1], Teresa Inés Moreno-Probance[4], Sebastian Ramos Onsins[5], Dominique Rocha[2*], Yuliaxis Ramayo-Caldas[2,6,7*]

[1]Facultad de Ciencias Agropecuarias, Universidad de Granma, Cuba

[2]GABI, INRA, AgroParisTech, Université Paris-Saclay, 78350, Jouy-en-Josas, France

[3]UR143, Unité de Recherches Zootechniques, Institut National de la Recherche Agronomique, Guadeloupe, France

[4]Empresa de Mejora Genética Manuel Fajardo, Jiguaní, Granma, Cuba

[5]Animal Genomics Department, Centre for Research in Agricultural Genomics (CRAG), Campus UAB, Bellaterra, 08193, Spain

[6]Animal Breeding and Genetics Program, Institute for Research and Technology in Food and Agriculture (IRTA), Torre Marimon, Caldes de Montbui, 08140, Spain

[7]Departament de Ciència Animal i dels Aliments, Universitat Autònoma de Barcelona (UAB), 08193, Bellaterra, Spain

Supplementary Table 1. Genomics regions with evidence of recent positive selection between Cuban (CHCU) and French Charolais (CHA).

Supplementary Table 2. Description of the ln(Rsb) values corresponding to SNPs within genomics regions with evidence of recent positive selection between Cuban (CHCU) and French Charolais (CHA).

Supplementary Table 3. Description of the over-represented canonical pathways in each breed.

Supplementary Table 4. Description of the samples used in the study.

Supplementary Figure 1. Phylogenetic tree representing the relationships between Bos taurus (red), Bos indicus (black) and Hybrid breeds (green).CHCU and CHA samples are indicated using blue and light blue color respectively.

Supplementary Figure 2. Ancestry models with ancestral populations (K=4, K=5, K=10, K=15, K=20, K=25, K=30, K=40). Breed names correspond to ABO: Abondance, AN: Angus, AUB: Aubrac, BALI: Bali, BAO: Baoule, BEFM: Beefmaster, BOR: Boran, BORG: Borgou, BR: Brahman, BRVH: Braunvieh, BSW: Brown Swiss, CANC: Canchim, CHA: French Charolais, CHCU: Cuban Charolais, GAS: Gascon, GEL: Gelbvieh, GIR: Gir, GNS: Guernsey, HFD: Hereford, HO: Holstein, JER: Jersey, KUR: Kuri, LAG: Lagune, LM: Limousin, MAAN: Maine-Anjou, MONT: Montbeliard, NDAM: N'Dama, NEL: Nelore, NORM: Normande, NRC: Norwegian Red, ONG: Ongole Grade, PIED: Piedmontese, PRP: French Red Pied Lowland, RMG: Romagnola, SAL: Salers, SGT: Santa Gertrudis, SIM: Simmental, SOM: Somba, TXLH: Texas Longhorn, VOS: Vosgienne, ZBO: Zebu Bororo, ZEB: East African Shorthorn Zebu, ZFU: Zebu Fulani, ZMA: Zebu from Madagascar.

Supplementary Table 1. Genomics regions with evidence of recent positive selection between Cuban (CHCU) and French Charolais (CHA).

Intervals	BTA	Start	End	Size (Mb)	# SNPs	Breed	Genes	Bibliographic reference
1	1	28906462	29095768	0.18	5	CHCU		[12,58–62]
2	1	39595785	39692931	0.09	3	CHCU		
3	1	60787763	60862725	0.07	3	CHCU	*GAP43*	
4	1	122814776	123672861	0.85	3	CHA	*PLOD2, PLSCR4, PLSCR5*	[58,63]
5	1	126606801	128447062	1.84	3	CHA	*ATP1B3, ATR, CHST2, GK5, GRK7, PAQR9, PCOLCE2, PLS1, RASA2, RNF7, SLC9A9, TFDP2, TRPC1, U2SURP, XRN1*	[61]
6	1	129007020	129388513	0.383	4	CHA	*CLSTN2, SLC25A36, TRIM42*	
7	2	28722493	28758897	0.03	2	CHCU		
8	2	76335841	77202062	0.861	6	CHA and CHCU	*CNTNAP5*	[12,57,60–62]
9	2	78942271	79158290	0.21	3	CHCU		
10	2	108437574	109812343	1.37	7	CHA		
11	3	33741850	34494339	0.75	6	CHCU	*GSTM3, AMPD2, GNAT2, GNAI3, GPR61, AMIGO1, CYB561D1, ATXN7L2, SYPL2, PSMA5, SORT1, MYBPHL, PSRC1, CELSR2, SARS, KIAA1324, C1orf194, TMEM167B, TAF13, WDR47*	[66]
12	3	88923355	89738009	0.81	6	CHA	*DAB1*	[11,12,62,67–69]
13	3	91524310	93184806	1.66	2	CHA	*ACOT11, BSND, CDCP2, DHCR24, FAM151A, HSPB11, LDLRAD1, LEXM, LRRC42, NDC1, PARS2, SSBP3, TCEANC2, TMEM59, TMEM61, TTC22, TTC4, USP24*	[11,12,60,62,63,67–70]
14	4	37385962	38257758	0.87	3	CHA	*PCLO, SEMA3E*	[12,70]
15	4	60118562	60809733	0.69	3	CHA	*ELMO1*	
16	4	73791282	74579013	0.78	3	CHA	*ZNF804B*	[63,71]
17	4	87770622	89708810	1.93	2	CHCU	*ASB15, CADPS2, HYAL4, IQUB, LMOD2, NDUFA5, SLC13A1, TAS2R16, WASL*	[12,72]
18	4	91051469	92588608	1.53	7	CHCU	*ZNF800*	[62,63]

19	4	99542142	100881943	1.33	2	CHCU	*AGBL3, C7orf49, C7orf73, CALD1, CNOT4, FAM180A, MTPN, NUP205, SLC13A4, STRA8, TMEM140, WDR91*	12,59,63,71–73
20	5	22020313	23461203	1.44	3	CHCU	*BTG1, C12orf74, EEA1, HIST1H2BL, MRPL42, PLEKHG7, UBE2N*	18
21	5	28660813	30114907	1.45	4	CHCU	*AQP2, AQP5, AQP6, ASIC1, ATF1, BIN2, CERS5, COX14, CSRNP2, DAZAP2, DIP2B, FAM186A, GPD1, HIGD1C, LARP4, LETMD1, LIMA1, RACGAP1, SMAGP, SMARCD1, TFCP2*	12,60,72
22	5	103860658	103911258	0.05	2	CHA	*ATN1, ENO2, LRRC23, PTPN6, C12orf57*	11,12,59,60,62,68,70,72,73
23	5	107167760	107362671	0.19	2	CHA	*TEAD4, TULP3*	
24	5	110300207	111836264	1.53	4	CHA	*ATF4, BAIAP2L2, CACNA1I, CBX6, CBX7, CBY1, DDX17, DMC1, ENTHD1, FAM227A, GRAP2, GTPBP1, JOSD1, KDELR3, MAFF, MGAT3, MIEF1, NPTXR, PDGFB, PICK1, PLA2G6, RPS19BP1, SLC16A8, SUN2, SYNGR1, TAB1, TMEM184B, TOM22*	59,60,73,74
25	6	4310838	4890621	0.57	3	CHA	*NDNF, PRDM5*	12,35,62,75
26	6	12804438	13145751	0.34	3	CHA	*CAMK2D*	12
27	6	23436503	24975034	1.53	2	CHA	*NFKB1, PPP3CA, SLC39A8*	
28	6	41795944	42387759	0.59	4	CHCU	*KCNIP4*	
29	6	44991839	45017700	0.02	2	CHCU		
30	6	79680793	79817258	0.13	2	CHCU		12,62,68–70,74
31	6	90966250	91485845	0.51	2	CHCU	*AREG, BTC, EPGN, EREG, MTHFD2L*	37,63
32	7	35071656	35099734	0.028	2	CHCU		
33	7	102535821	104185842	1.65	4	CHA	*SLCO4C1, SLCO6A1, ST8SIA4*	
34	7	107837688	109751820	1.91	4	CHA	*EFNA5*	
35	8	18759713	20564440	1.80	3	CHA and CHCU	*ELAVL2, IZUMO3*	
36	8	21073477	21197694	0.12	4	CHA and CHCU		
37	8	24188422	24365893	0.17	2	CHA		63
38	8	42460982	42486699	0.02	2	CHCU		12,19,60,62,63,75,76
39	8	97338128	98746331	1.41	2	CHCU	*RAD23B, ZNF462*	
40	8	99957737	101231366	1.27	2	CHCU	*ACTL7A, ACTL7B, CTNNAL1, EPB41L4B, FAM206A, FRRS1L, IKBKAP, MUSK, PALM2, PTPN3, SVEP1, TMEM245*	

41	9	2763068	3158770	0.39	3	CHCU		
42	9	13945212	15820879	1.87	3	CHCU	*COL12A1, COX7A2, FILIP1, MYO6, SENP6, TMEM30A*	20
43	9	49449716	51395314	1.94	7	CHCU	*ASCC3, CCNC, COQ3, FAXC, MCHR2, PNISR, PRDM13, SIM1, USP45*	12,59,62,63,72,73,75,77
44	9	90080368	90155533	0.075	2	CHA	*ESR1*	12,59,61–63,70,73
45	10	33736444	34042986	0.30	2	CHA	*SPRED1*	
46	10	44006781	44556318	0.54	3	CHA	*FRMD6, TMX1*	61,73,74
47	10	85187766	87108872	1.92	3	CHA	*ABCD4, ACYP1, ALDH6A1, AREL1, BBOF1, C14orf169, COQ6, DLST, DNAL1, EIF2B2, ELMSAN1, ENTPD5, FAM161B, FCF1, FOS, HEATR4, ISCA2, JDP2, LIN52, LTBP2, MLH3, NEK9, NPC2, PGF, PNMA1, PROX2, RPL18A, RPS6KL1, SYNDIG1L, TMED10, VRTN, VSX2, YLPM1, ZC2HC1C, ZNF410*	
48	10	90725565	91580758	0.85	5	CHCU		19
49	11	1920092	3736020	1.81	5	CHCU	*ACTR1B, ADRA2B, ANKRD23, ANKRD39, ARID5A, ASTL, CIAO1, CNGA3, CNNM3, CNNM4, DUSP2, FAM178B, FER1L5, GPAT2, INPP4A, ITPRIPL1, KANSL3, PROM2, SEMA4C, SNRNP200, STARD7, TMEM127, TMEM131, VWA3B, ZAP70, ZNF2, ZNF514 NCAPH, NEURL3, LMAN2L*	12,61,63,68,72,73
50	11	7482969	7540560	0.057	2	CHA	*TMEM182*	
51	11	57276682	57296795	0.02	2	CHCU		
52	11	72973534	74079196	1.10	2	CHCU	*ADGRF3, ASXL2, C2orf70, DNMT3A, DRC1, DTNB, EPT1, GAREM2, HADHA, HADHB, KIF3C, RAB10, OTOF*	
53	11	77735570	78708174	0.97	4	CHCU	*APOB, GDF7, HS1BP3, LDAH, PUM2, RHOB, SDC1*	12,19,72
54	12	3279272	4685516	1.40	4	CHA		19
55	12	16167243	16756600	0.58	3	CHA	*CPB2, KIAA0226L, LCP1, LRCH1, LRRC63, ZC3H13*	12,58,63,71,72,78
56	12	68657690	68728376	0.07	2	CHCU		

57	13	42221994	42312878	0.09	2	CHCU	*CD93*	11,12,61,73,77
58	13	63184499	63257337	0.07	2	CHCU	*BPIFA1, BPIFA3*	12,61,62,65,67,68,70,72
59	13	73681829	75383374	1.70	2	CHA	*PKIG, ADA, KCNK1, RIMS4, YWHAB, PABPC1L, TOMM34, STK4, KCNS1, WFDC5, SLPI, MATN4, RBPJL, SDC4, SYS1, TP53TG5, DBNDD2, PIGT, PTI, WFDC2, WFDC8, WFDC10A, WFDC11, WFDC10B, WFDC13, SPINT4, DNTTIP1, UBE2C, TNNC2, ACOT8, ZSWIM3, ZSWIM1, SPATA25, NEURL2, CTSA, PLTP, ZMYND8, NCOA3, SULF2*	62,63
60	13	76616107	77076450	0.46	2	CHA	*NCOA3, SULF2, ZMYND8*	
61	14	10051740	10424817	0.37	5	CHCU	*HHLA1, KCNQ3*	11,12,60,63
62	14	27231200	27271835	0.04	2	CHA		11,12,60,62,63,69,71,75,77
63	14	57340508	57584280	0.24	3	CHA	*NUDCD1, TRHR*	60,63
64	15	23367462	23620358	0.25	3	CHCU		
65	15	26138126	26697713	0.55	3	CHA	*CADM1*	
66	15	53622495	54769019	1.14	3	CHCU	*ARHGEF17, RELT, FAM168A, PLEKHB1, MRPL48, DNAJB13, UCP2, UCP3, C2CD3, PPME1, P4HA3, PGM2L1, LIPT2, POLD3, H2AFX, RNF26, KCNE3*	
67	15	72771875	72914738	0.14	2	CHA		12,62,75,77
68	16	21498689	22179895	0.68	5	CHA	*GPATCH2*	12,60,71–73,76
69	16	30626532	31654996	1.02	3	CHCU	*ADCK3, AHCTF1, CDC42BPA, CNST, SCCPDH, TFB2M, SMYD3*	63
70	16	33003888	33641966	0.63	3	CHCU	*ADSS, C1orf100, C1orf101, COX20, DESI2, EFCAB2, HNRNPU*	63
71	17	30813823	30870156	0.05	2	CHA		
72	17	47808938	47926452	0.11	3	CHCU		
73	17	57045290	57068849	0.02	2	CHA	*CUX2*	12,62,63,72,73
74	17	67245920	67626935	0.38	2	CHCU	*CRYBB2, CRYBB3, KIAA1671, KIAA1671, PIWIL3, SGSM1, TMEM211*	
75	18	1983526	2108272	0.12	2	CHA	*GLG1, TPT1*	12,35,68

76	18	16594926	16671415	0.07	2	CHA	*ABCC11, ABCC12*	11,60
77	18	25743634	27374063	1.63	4	CHCU	*ADGRG3, CCDC113, CFAP20, CNGB1, CNOT1, CSNK2A2, DRC7, GINS3, GOT2, KATNB1, KIFC3, MMP15, NDRG4, PRSS54, SETD6, SLC38A7, TEPP, USB1, ZNF319*	12,63,68,76
78	19	7461446	7511302	0.049	2	CHCU	*ANKFN1*	
79	19	9079748	9115265	0.03	2	CHCU		37
80	19	17888348	17964542	0.07	2	CHA	*ASIC2*	
81	20	53674655	54239646	0.56	2	CHCU	*CDH18*	
82	20	69615449	70062996	0.44	4	CHCU	*AHNAK2, IGHE, CRIP1, CRIP2*	35
83	21	21831416	22435618	0.60	2	CHCU	*BLM, CIB1, CRTC3, FES, FURIN, GDPGP1, HDDC3, IDH2, MAN2A2, NGRN, PRC1, RCCD1, SEMA4B, UNC45A, VPS33B, ZNF710*	
84	21	25036501	25704052	0.66	3	CHA	*BNC1, BTBD1, CTSH, HDGFRP3, TM6SF1*	79
85	21	35564875	36136065	0.57	3	CHA and CHCU	*STXBP6*	
86	22	6034078	6876710	0.84	3	CHA	*CMTM8, GPD1L, OSBPL10, STT3B*	
87	22	10476965	11159199	0.68	5	CHCU	*TRAK1, DCLK3, GOLGA4, ITGA9, EPM2AIP1, LRRFIP2, MLH1*	12,19,35,59,61–63,70,72
88	23	6999212	7227396	0.22	3	CHCU	*HLA-DMA, HLA-DOA, HLA-DOB, PSMB8, PSMB9, TAP1, TAP2*	11
89	23	23909884	24989231	1.07	4	CHCU	*EFHC1, GSTA4, ICK, IL17, IL17F, MCM3, PAQR8, PKHD1, TMEM14A, TRAM2*	11,12,60,63,72
90	24	9058158	10890316	1.83	2	CHA	*DSEL, CDH19*	
91	24	19306436	19498323	0.19	2	CHCU		
92	24	22837383	23027518	0.19	3	CHCU		20,72,80
93	24	60613517	61001528	0.38	2	CHCU	*PIGN*	63,73,78
94	25	7381787	8165854	0.78	3	CHA	*ABAT, C16orf72, CARHSP1, METTL22, PMM2, TMEM114, TMEM186, USP7*	
95	25	34800159	36383338	1.58	3	CHCU	*ACHE, ALKBH4, AP1S1, CLDN15, COL26A1, CUX1, DTX2, EPHB4, FIS1, HSPB1, IFT22, LRWD1, MYL10, ORAI2, PLOD3, POLR2J, PRKRIP1, SERPINE1, SH2B2, SLC12A9, SRRM3, SRRT, SSC4D, TRIM56, TRIP6, UFSP1, UPK3B, VGF, YWHAG, ZAN, ZNHIT1*	12,62,72,73

96	26	12102814	12295284	0.19	3	CHCU		
97	26	48223351	48300208	0.07	2	CHCU		19
98	27	25529207	25606547	0.07	2	CHA		
99	27	43038925	43104231	0.06	2	CHCU		
100	28	34855538	36300699	1.44	3	CHCU	*ANXA11, DYDC1, DYDC2, FAM213A, MAT1A, PLAC9, PPIF, SFTPA1, SH2D4B, TMEM254, TSPAN14, ZCCHC24, ZMIZ1*	
101	28	42651360	43667216	1.01	3	CHA	*ARHGAP22, FRMPD2, LRRC18, MAPK8, PTPN20, WDFY4*	12,63,72
102	29	14116468	14186603	0.07	2	CHA		
103	29	25110723	25143584	0.03	2	CHCU	*NAV2*	
104	29	37913830	37949466	0.03	2	CHA	*CD6*	12,63,70,72,73

Suplementary Table 2. Description of the ln(Rsb) values corresponding to SNPs within genomics regions with evidence of recent positive selection between Cuban (CHCU) and French Charolais (CHA).

SNP	Chr	BP	ln(Rsb)
BTB-00009232	1	25607948	-2.891
ARS-BFGL-NGS-119690	1	28906462	-2.836
BTB-00012425	1	28978223	-2.992
BTB-00012448	1	29004968	-3.248
BTB-01734642	1	29073969	-3.151
ARS-BFGL-NGS-108911	1	29095768	-3.288
Hapmap51428-BTA-26864	1	39595785	-3.035
BTB-00018760	1	39653311	-2.910
Hapmap38790-BTA-87413	1	39692931	-3.362
BTA-23353-no-rs	1	60787763	-3.060
ARS-BFGL-NGS-117152	1	60822626	-2.793
UA-IFASA-7336	1	60862725	-3.147
ARS-BFGL-NGS-105510	1	122814776	2.689
ARS-BFGL-BAC-14851	1	123646279	2.714
Hapmap38963-BTA-50274	1	123672861	2.918
ARS-BFGL-NGS-113021	1	126606801	2.901
ARS-BFGL-NGS-34197	1	128420783	3.114
ARS-BFGL-NGS-111607	1	128447062	3.155
BTB-00057251	1	129007020	3.063
ARS-BFGL-NGS-24528	1	129101492	3.237
ARS-BFGL-NGS-14969	1	129339868	3.273
ARS-BFGL-NGS-116542	1	129388513	3.270
ARS-BFGL-NGS-91441	1	141691093	-2.932
BTA-93171-no-rs	2	13112798	-2.431
BTB-01728391	2	21267547	-2.543
Hapmap41697-BTA-96617	2	28722493	-2.513
BTA-96624-no-rs	2	28758897	-2.498
BTB-01063267	2	76335841	-2.180
Hapmap50277-BTA-20931	2	76597004	2.720
ARS-BFGL-NGS-106237	2	76637668	2.593
Hapmap42472-BTA-21372	2	76675977	2.828
BTB-00917603	2	76730474	-2.705
BTB-00103137	2	77202062	-2.197
Hapmap61010-rs29023283	2	78942271	-2.322
BTB-01809666	2	79090198	-2.617
UA-IFASA-2241	2	79158290	-2.628
Hapmap49925-BTA-24427	2	86704498	-2.413
BTB-00109619	2	108437574	2.804
ARS-BFGL-NGS-72876	2	108464952	2.925
BTB-00109560	2	108486036	2.808
ARS-BFGL-NGS-99327	2	108529237	3.303
ARS-BFGL-NGS-14681	2	108549488	2.643

Hapmap49392-BTA-97343	2	108843619	3.032
Hapmap35128-BES3_Contig428_1619	2	109812343	2.619
ARS-BFGL-NGS-60573	2	134388958	2.759
ARS-BFGL-NGS-6776	3	16279733	-2.179
ARS-BFGL-NGS-86767	3	33741850	-2.745
ARS-BFGL-NGS-115128	3	33895762	-2.315
Hapmap42629-BTA-67307	3	33948546	-2.096
Hapmap41054-BTA-67528	3	34344799	-2.403
BTA-67533-no-rs	3	34439644	-2.594
Hapmap41457-BTA-121582	3	34494339	-2.420
BTB-01405537	3	40364628	-2.340
ARS-BFGL-NGS-117463	3	86907103	3.985
Hapmap41488-BTA-24106	3	88923355	4.430
BTA-11699-no-rs	3	89151787	4.273
BTB-00143286	3	89233653	4.478
BTB-00143272	3	89257029	4.023
ARS-BFGL-NGS-100813	3	89320961	3.819
BTB-00142497	3	89738009	3.965
BTB-01195369	3	91524310	4.040
ARS-BFGL-NGS-65964	3	93184806	3.951
BTB-00153367	3	102775859	3.837
Hapmap60225-rs29010786	4	37385962	3.773
Hapmap38426-BTA-70226	4	38213706	3.249
BTB-00178261	4	38257758	3.537
ARS-BFGL-NGS-25928	4	47697368	3.446
Hapmap51420-BTA-20855	4	60118562	3.317
ARS-BFGL-NGS-115377	4	60781458	3.510
ARS-BFGL-NGS-46170	4	60809733	3.385
BTB-00197052	4	73791282	3.511
BTB-02044451	4	74124684	3.714
BTB-00197584	4	74579013	3.500
BTB-01290382	4	87770622	-2.492
BTB-01323835	4	89708810	-2.446
BTA-109007-no-rs	4	91051469	-2.427
BTB-01845975	4	91190158	-2.525
Hapmap54638-rs29022155	4	91227550	-2.525
BTA-11696-no-rs	4	91417417	-2.432
ARS-BFGL-NGS-118100	4	92141993	-2.557
BTB-00201719	4	92176712	-2.670
ARS-BFGL-NGS-94823	4	92588608	-3.362
BTA-22968-no-rs	4	99542142	-2.430
BTA-71976-no-rs	4	100881943	-2.421
BTA-72912-no-rs	5	22020313	-2.686
ARS-BFGL-NGS-12094	5	22053661	-2.923
BTA-72955-no-rs	5	23461203	-2.749
ARS-BFGL-NGS-10291	5	24512405	-2.789
ARS-BFGL-NGS-107085	5	28660813	-2.949
ARS-BFGL-NGS-49972	5	30012017	-2.875
Hapmap34759-BES10_Contig780_1565	5	30061770	-2.586
Hapmap47089-BTA-73292	5	30114907	-2.800
ARS-BFGL-NGS-678	5	103860658	2.694

ARS-BFGL-NGS-18320	5	103911258	3.016
ARS-BFGL-NGS-37722	5	107167760	2.927
Hapmap57466-rs29018274	5	107362671	2.661
ARS-BFGL-NGS-61240	5	110300207	2.848
ARS-BFGL-NGS-86565	5	111420622	2.654
ARS-BFGL-NGS-43376	5	111790193	2.726
BTA-75067-no-rs	5	111836264	2.746
Hapmap59748-rs29024200	5	118065604	3.003
ARS-BFGL-NGS-77840	6	4310838	2.282
BTB-01767757	6	4670459	2.179
BTB-01468045	6	4890621	2.335
BTB-00103730	6	12804438	2.166
BTA-22128-no-rs	6	13040713	2.202
Hapmap47406-BTA-77006	6	13145751	2.380
BTB-00246860	6	23436503	2.333
BTA-37417-no-rs	6	24975034	2.269
BTB-00250665	6	41795944	-3.209
Hapmap48459-BTA-75920	6	42023749	-2.701
Hapmap49744-BTA-75903	6	42120804	-2.810
BTB-00251852	6	42387759	-2.768
Hapmap23242-BTC-039071	6	44991839	-2.911
BTB-00254199	6	45017700	-2.814
ARS-BFGL-NGS-104919	6	72259859	-2.811
ARS-BFGL-NGS-112473	6	79680793	-2.746
BTB-01900612	6	79817258	-2.812
Hapmap49297-BTA-76961	6	83147633	-2.747
ARS-BFGL-NGS-116877	6	90966250	2.201
BTB-01657893	6	91485845	2.250
Hapmap51146-BTA-112717	7	35071656	-1.847
BTA-112718-no-rs	7	35099734	-1.935
UA-IFASA-9367	7	41114885	-1.916
BTB-00313206	7	60055026	-1.935
ARS-BFGL-NGS-89122	7	65636193	3.436
ARS-BFGL-NGS-37793	7	74773447	3.583
Hapmap50786-BTA-80052	7	82209855	3.475
ARS-BFGL-NGS-80444	7	84991860	3.776
Hapmap60739-rs29026571	7	90923765	3.579
UA-IFASA-5377	7	102535821	3.629
BTA-80429-no-rs	7	102564643	3.514
ARS-BFGL-NGS-118583	7	104072416	3.596
Hapmap43948-BTA-80452	7	104185842	3.601
BTB-01553536	7	107837688	-1.775
Hapmap39686-BTA-80533	7	109268080	-1.979
BTB-00330149	7	109293823	-1.992
ARS-BFGL-NGS-21760	7	109751820	-1.924
BTB-00332255	7	111510538	-1.840
ARS-BFGL-NGS-26055	8	18759713	-3.310
ARS-BFGL-NGS-26796	8	20439428	3.169
Hapmap53914-rs29021936	8	20564440	3.149
BTA-17775-no-rs	8	21073477	-3.116
BTA-118350-no-rs	8	21127450	-2.605

BTB-01751208	8	21167011	-2.789
ARS-BFGL-NGS-38473	8	21197694	3.483
BTA-103194-no-rs	8	24188422	3.184
ARS-BFGL-NGS-41681	8	24365893	3.419
BTB-00285653	8	30036807	3.167
BTA-80950-no-rs	8	36666306	3.688
Hapmap47481-BTA-105587	8	42460982	3.401
BTB-01581660	8	42486699	3.362
ARS-BFGL-NGS-114052	8	47041865	-2.488
Hapmap53171-rs29012580	8	97338128	-2.434
ARS-BFGL-NGS-24293	8	98746331	-2.502
Hapmap58894-rs29013940	8	99957737	-2.439
ARS-BFGL-NGS-111788	8	101231366	-2.469
Hapmap30835-BTA-146159	8	102104126	-2.692
BTB-01573029	9	2763068	-2.489
ARS-BFGL-NGS-29404	9	2934964	-2.568
ARS-BFGL-NGS-22429	9	3158770	-3.085
BTA-115251-no-rs	9	13945212	-2.662
BTB-01653669	9	14489468	-2.476
BTB-00380334	9	15820879	-2.677
Hapmap41217-BTA-27517	9	49449716	2.559
BTB-01347067	9	50208711	2.073
Hapmap54273-rs29023608	9	50591670	2.432
BTB-00391825	9	50632294	2.361
BTB-00391835	9	50652338	2.606
BTB-01931984	9	51243021	2.092
Hapmap46961-BTA-17297	9	51395314	2.139
BTA-84163-no-rs	9	72115536	2.080
BTB-00404625	9	90080368	-2.579
Hapmap42341-BTA-84711	9	90155533	-2.575
ARS-BFGL-NGS-20741	9	103270667	2.088
ARS-BFGL-NGS-3160	10	28548872	2.335
Hapmap43516-BTA-22426	10	28751801	2.674
BTA-62303-no-rs	10	28838152	2.617
ARS-BFGL-NGS-118622	10	33736444	2.128
Hapmap50429-BTA-64384	10	34042986	2.082
BTB-01885735	10	44006781	2.099
BTB-00979154	10	44521573	2.057
Hapmap42628-BTA-67218	10	44556318	2.117
BTB-00430147	10	55993255	2.444
BTB-00441121	10	85187766	-3.399
Hapmap57084-ss46526565	10	86654970	-3.278
ARS-BFGL-NGS-52747	10	87108872	-4.330
BTB-00443654	10	90725565	-3.408
Hapmap24141-BTA-125889	10	90747018	-3.847
ARS-BFGL-NGS-43349	10	91304831	-3.260
ARS-BFGL-NGS-112358	10	91398665	-3.311
Hapmap60776-rs29026927	10	91580758	-3.258
BTB-01101887	10	92846879	-3.306
ARS-BFGL-NGS-24329	11	1920092	3.241
Hapmap54920-rs29026446	11	1963074	3.138

ARS-BFGL-NGS-33034	11	2637529	3.318
BTB-01823731	11	3215052	3.732
BTB-00453425	11	3736020	3.170
Hapmap48515-BTA-89384	11	4799594	3.392
ARS-BFGL-NGS-28646	11	7482969	3.304
ARS-BFGL-NGS-37576	11	7540560	3.178
ARS-BFGL-NGS-15269	11	29578421	-2.418
Hapmap60754-rs29010392	11	57276682	-2.632
ARS-BFGL-BAC-12421	11	57296795	-2.900
ARS-BFGL-NGS-74702	11	72973534	-3.183
ARS-BFGL-NGS-102717	11	74079196	-2.376
UA-IFASA-4453	11	77735570	-2.674
ARS-BFGL-NGS-117322	11	77884744	-2.817
ARS-BFGL-NGS-91942	11	78069787	-2.481
ARS-BFGL-NGS-85521	11	78708174	-2.444
BTA-118578-no-rs	11	91546547	3.225
ARS-BFGL-NGS-45741	12	2766536	2.402
ARS-BFGL-NGS-32075	12	3279272	1.720
BTB-01713600	12	4484840	1.737
ARS-BFGL-NGS-114666	12	4653564	2.101
BTB-00485379	12	4685516	2.387
ARS-BFGL-NGS-23454	12	6960393	1.754
Hapmap58297-rs29023722	12	16167243	-3.442
BTA-31743-no-rs	12	16732810	-3.692
ARS-BFGL-NGS-90717	12	16756600	-3.907
ARS-BFGL-NGS-76809	12	29238096	-3.475
BTA-21678-no-rs	12	45196682	2.007
ARS-BFGL-NGS-30445	12	48488970	-3.430
ARS-BFGL-BAC-5848	12	68657690	-3.645
BTB-00500935	12	68728376	-3.682
BTB-01324017	13	1966648	2.100
ARS-BFGL-NGS-21977	13	30235320	2.212
BTB-00523106	13	42221994	-2.388
BTB-00523176	13	42312878	-2.377
ARS-BFGL-NGS-55042	13	45510831	-2.463
ARS-BFGL-NGS-41818	13	47546608	-2.429
ARS-BFGL-NGS-110904	13	59072057	2.324
ARS-BFGL-NGS-52692	13	63184499	2.565
BTA-33168-no-rs	13	63257337	2.162
Hapmap51212-BTA-33858	13	73681829	-2.342
ARS-USMARC-Parent-AY853303-no-rs	13	75383374	-2.549
BTB-00893866	13	76616107	2.122
ARS-BFGL-NGS-107515	13	77076450	2.669
Hapmap40030-BTA-87432	13	84053901	-2.937
ARS-BFGL-NGS-6240	14	5048719	-2.829
ARS-BFGL-NGS-110321	14	10051740	-2.832
ARS-BFGL-NGS-41494	14	10118287	-3.190
Hapmap24065-BTC-072634	14	10213875	-2.799
Hapmap26301-BTC-055949	14	10387828	-2.741
Hapmap25450-BTC-055819	14	10424817	-2.734
Hapmap23255-BTC-073028	14	27231200	2.015

Hapmap27112-BTC-063342	14	27271835	2.015
ARS-BFGL-BAC-23417	14	47823469	-3.046
ARS-BFGL-BAC-24084	14	57340508	1.964
ARS-BFGL-NGS-105323	14	57489773	2.159
Hapmap24398-BTC-062971	14	57584280	1.969
ARS-BFGL-NGS-1433	14	71433555	2.350
UA-IFASA-6510	14	77853126	2.170
BTB-00584953	15	23367462	-2.745
BTA-38113-no-rs	15	23593850	-3.027
ARS-BFGL-NGS-89413	15	23620358	-3.142
BTB-01138108	15	26138126	-2.617
ARS-BFGL-NGS-114536	15	26660750	-3.124
ARS-BFGL-NGS-27798	15	26697713	-2.860
ARS-BFGL-NGS-86583	15	30076410	-2.997
Hapmap35547-SCAFFOLD316738_25851	15	53622495	2.798
Hapmap40723-BTA-37075	15	54301900	2.782
ARS-BFGL-NGS-17983	15	54769019	2.709
Hapmap45874-BTA-37175	15	58830757	3.020
Hapmap44538-BTA-37377	15	66208675	3.133
Hapmap34688-BES2_Contig423_849	15	72771875	2.853
Hapmap42526-BTA-37604	15	72914738	3.752
Hapmap49982-BTA-39672	16	10335068	-2.359
BTB-00629361	16	21498689	-2.480
BTB-00629893	16	21772991	-2.489
ARS-BFGL-NGS-55380	16	21821449	-2.687
BTA-40382-no-rs	16	22101902	-2.312
ARS-BFGL-NGS-89535	16	22179895	-2.332
ARS-BFGL-NGS-68395	16	30626532	2.028
ARS-BFGL-NGS-37475	16	31499868	1.981
BTB-00633646	16	31654996	2.007
BTB-00634325	16	33003888	2.004
BTB-00634312	16	33121540	1.964
Hapmap40684-BTA-16356	16	33641966	2.223
Hapmap46938-BTA-114095	16	69795545	-2.475
ARS-BFGL-NGS-66923	17	18910914	-2.855
BTB-01839787	17	30813823	2.438
BTB-01757364	17	30870156	2.324
Hapmap34868-VSWHP1E1013A_scf	17	33948813	2.709
ARS-BFGL-NGS-104812	17	47808938	-2.677
ARS-BFGL-NGS-9657	17	47898312	-2.865
ARS-BFGL-NGS-22143	17	47926452	-2.698
ARS-BFGL-NGS-102781	17	48084367	-2.960
Hapmap43572-BTA-41227	17	57045290	-3.373
ARS-BFGL-NGS-117067	17	57068849	-3.014
BTB-00686912	17	67245920	2.662
Hapmap48532-BTA-95668	17	67626935	2.382
Hapmap44075-BTA-41915	17	72384103	2.218
ARS-BFGL-BAC-36904	18	1983526	1.846
BTA-44477-no-rs	18	2108272	2.180
ARS-BFGL-NGS-41174	18	16594926	-2.264

BTA-97570-no-rs	18	16671415	-2.308
ARS-BFGL-NGS-19178	18	22610574	-2.446
ARS-BFGL-BAC-2323	18	25743634	1.935
ARS-BFGL-NGS-80672	18	26630875	1.849
Hapmap23161-BTA-162019	18	27281676	2.404
ARS-BFGL-NGS-29803	18	27374063	2.153
ARS-BFGL-NGS-92089	18	33542286	-2.510
ARS-BFGL-NGS-33879	18	48172057	-2.490
UA-IFASA-8401	19	7461446	-2.252
Hapmap40983-BTA-44816	19	7511302	-2.113
Hapmap42218-BTA-45703	19	9079748	-2.148
ARS-BFGL-NGS-6594	19	9115265	-2.146
ARS-BFGL-NGS-6764	19	16750367	2.376
ARS-BFGL-NGS-112779	19	17888348	2.891
Hapmap39489-BTA-44619	19	17964542	2.478
ARS-BFGL-NGS-77060	19	50421201	2.724
ARS-BFGL-NGS-44006	19	57592897	-2.179
UA-IFASA-8231	19	60274670	-2.071
Hapmap51158-BTA-117076	20	199074	2.285
ARS-BFGL-NGS-107987	20	2452442	2.225
ARS-BFGL-NGS-113565	20	24861992	2.047
ARS-BFGL-NGS-65409	20	39275917	-2.895
BTB-00786292	20	46035562	2.437
BTA-50817-no-rs	20	53674655	1.983
BTA-92600-no-rs	20	54239646	2.047
BTA-96274-no-rs	20	63519841	-2.638
ARS-BFGL-NGS-28691	20	67060157	-2.939
Hapmap40393-BTA-112315	20	69615449	-3.208
ARS-BFGL-NGS-114933	20	69916426	-2.930
BTA-51296-no-rs	20	69974084	-2.775
BTB-00799796	20	70062996	-3.140
Hapmap41293-BTA-53465	21	12712258	2.928
ARS-BFGL-NGS-84831	21	21831416	3.001
Hapmap43603-BTA-51704	21	22435618	2.976
BTA-51880-no-rs	21	25036501	-2.793
ARS-BFGL-NGS-109972	21	25057520	-2.741
Hapmap53212-rs29015272	21	25704052	-2.603
BTB-00812805	21	27477084	2.958
Hapmap50721-BTA-52111	21	35564875	-2.969
ARS-BFGL-NGS-29881	21	35640578	-3.367
ARS-BFGL-NGS-92434	21	36136065	3.041
Hapmap42788-BTA-107220	21	48808007	-2.799
Hapmap32052-BTA-136158	21	65272069	3.138
Hapmap36468-SCAFFOLD225014_6126	22	3200170	1.475
BTB-00832694	22	6034078	-2.951
Hapmap61006-rs29024592	22	6140185	-2.866
ARS-BFGL-NGS-1271	22	6876710	-2.287
BTA-55229-no-rs	22	10476965	1.647
ARS-BFGL-NGS-115144	22	10657184	1.565
BTB-01184669	22	10998835	1.364
Hapmap50552-BTA-106670	22	11063911	1.494

BTA-55272-no-rs	22	11159199	1.357
ARS-BFGL-NGS-21410	22	17556764	-2.307
ARS-BFGL-NGS-35996	22	28161068	-2.397
Hapmap40908-BTA-121388	23	6999212	2.226
Hapmap60475-rs29022896	23	7191371	2.162
BTA-57090-no-rs	23	7227396	2.370
UA-IFASA-8890	23	23909884	-2.265
Hapmap30807-BTA-137195	23	24053849	-2.539
ARS-BFGL-BAC-5865	23	24117682	-2.894
ARS-BFGL-NGS-44911	23	24989231	-2.601
ARS-BFGL-NGS-43531	23	42550545	1.984
Hapmap54014-rs29018901	24	541784	2.423
Hapmap49224-BTA-57251	24	9058158	2.183
BTB-01140405	24	10890316	2.554
ARS-BFGL-NGS-5419	24	19306436	-2.971
ARS-BFGL-NGS-38991	24	19498323	-2.855
Hapmap59298-rs29022301	24	22837383	-3.079
BTA-57610-no-rs	24	22862463	-3.079
ARS-BFGL-NGS-91126	24	23027518	-3.258
ARS-BFGL-NGS-103861	24	60613517	2.281
Hapmap40009-BTA-58757	24	61001528	2.280
Hapmap31636-BTC-053953	25	4544289	-2.721
ARS-BFGL-NGS-3547	25	7381787	-3.332
Hapmap42268-BTA-60619	25	7393865	-3.131
ARS-BFGL-NGS-113918	25	8165854	-2.706
Hapmap22767-BTC-029613	25	30116355	2.519
ARS-BFGL-NGS-71416	25	34800159	2.279
ARS-BFGL-NGS-107782	25	35657709	2.672
Hapmap52775-rs29010405	25	36383338	2.571
BTB-00925415	26	11102722	1.959
Hapmap46894-BTA-89312	26	12102814	-2.770
ARS-BFGL-NGS-12381	26	12200948	-2.773
BTB-01841682	26	12295284	-3.056
Hapmap44206-BTA-119750	26	30168678	-2.830
ARS-BFGL-NGS-80584	26	33153900	1.620
ARS-BFGL-NGS-84251	26	39452485	-2.896
ARS-BFGL-NGS-118475	26	48223351	1.418
BTA-61880-no-rs	26	48300208	1.436
ARS-BFGL-NGS-113226	27	97306	-2.276
BTB-01738922	27	15202709	1.744
BTB-00964618	27	25529207	1.974
Hapmap43311-BTA-62552	27	25606547	1.647
BTB-01759195	27	32513708	2.011
ARS-BFGL-NGS-73826	27	38053835	-1.646
BTB-01460972	27	43038925	-1.776
ARS-BFGL-NGS-79964	27	43104231	-1.715
ARS-BFGL-NGS-81213	28	7715648	-2.003
Hapmap51751-BTA-63790	28	23500352	2.799
ARS-BFGL-NGS-29739	28	34855538	2.904
ARS-BFGL-NGS-15751	28	35076001	2.936
ARS-BFGL-NGS-33494	28	36300699	3.265

ARS-BFGL-NGS-34198	28	42651360	-1.822
BTA-64609-no-rs	28	43217773	-2.116
Hapmap36705-SCAFFOLD40294_36721	28	43667216	-2.347
ARS-BFGL-NGS-33829	29	11940671	2.798
BTB-01354660	29	14116468	2.430
BTB-01354776	29	14186603	2.475
ARS-BFGL-NGS-56020	29	25110723	-1.836
Hapmap41325-BTA-65112	29	25143584	-1.744
UA-IFASA-8767	29	32916926	2.450
ARS-BFGL-NGS-43147	29	37913830	-2.341
ARS-BFGL-NGS-33015	29	37949466	-2.541

Supplementary Table 4. Description of the samples used in the study.

Breed	Breed Code	Sub Species	Continent	Geographic Origin
Abondance	ABO	Bos taurus	Europe	Southeast France
Angus	AN	Bos taurus	Europe	Aberdeenshire, Scotland
Aubrac	AUB	Bos taurus	Europe	South France
Baoule	BAO	Bos taurus	Africa	Burkina Faso
Beefmaster	BEFM	Hybrid	Americas	Texas, United States
Boran	BOR	Hybrid	Africa	southern Ethiopia
Borgou	BORG	Hybrid	Africa	Benin
Brahman	BR	Bos indicus	Americas	Gulf Coast, United States
Braunvieh	BRVH	Bos taurus	Europe	Switzerland
Brown Swiss	BSW	Bos taurus	Americas	United States
Canchim	CANC	Hybrid	Americas	Brazil
Charolais	CHA	Bos taurus	Europe	Saône-et-Loire, France
East African Shorthorn Zebu	ZEB	Hybrid	Africa	Kenya
French Red Pied Lowland	PRP	Bos taurus	Europe	Northwest France
Gascon	GAS	Bos taurus	Europe	Southwest France
Gelbvieh	GEL	Bos taurus	Europe	Central Germany
Gir	GIR	Bos indicus	Asia	Gujerat, India
Guernsey	GNS	Bos taurus	Europe	Guernsey Island
Hereford	HFD	Bos taurus	Europe	Wales
Holstein	HO	Bos taurus	Europe	Holland
Jersey	JER	Bos taurus	Europe	Jersey Island
Kuri	KUR	Bos taurus	Africa	Chad
Lagune	LAG	Bos taurus	Africa	Benin
Limousin	LM	Bos taurus	Europe	Massif Central, France
Maine-Anjou	MAAN	Bos taurus	Europe	Brittany, France
Montbeliard	MONT	Bos taurus	Europe	France
N'Dama	NDAM	Bos taurus	Africa	Ivory Coast, Africa
Nelore	NEL	Bos indicus	Americas	Brazil
Normande	NORM	Bos taurus	Europe	France
Norwegian Red	NRC	Bos taurus	Europe	Norway
Ongole Grade	ONG	Bos indicus	Asia	Andhra Pradesh, India
Piedmontese	PIED	Bos taurus	Europe	Northwest Italy
Romagnola	RMG	Bos taurus	Europe	Emilia, Italy
Salers	SAL	Bos taurus	Europe	Massif Central, France
Santa Gertrudis	SGT	Hybrid	Americas	Texas, United States
Simmental	SIM	Bos taurus	Europe	Bern, Switzerland
Somba	SOM	Bos taurus	Africa	Togo
Texas Longhorn	TXLH	Bos taurus	Americas	Texas, United States
Vosgienne	VOS	Bos taurus	Europe	Northeast France
Zebu Bororo	ZBO	Bos indicus	Africa	Chad
Zebu from Madagascar	ZMA	Bos indicus	Africa	Madagascar
Zebu Fulani	ZFU	Bos indicus	Africa	Benin

Supplementary Figure 1. Phylogenetic tree representing the relationships between Bos taurus (red), Bos indicus (black) and Hybrid breeds (green).CHCU and CHA samples are indicated using blue and light blue color respectively. The robustness of the tree was supported by bootstrap (100 bootstraps) analysis.

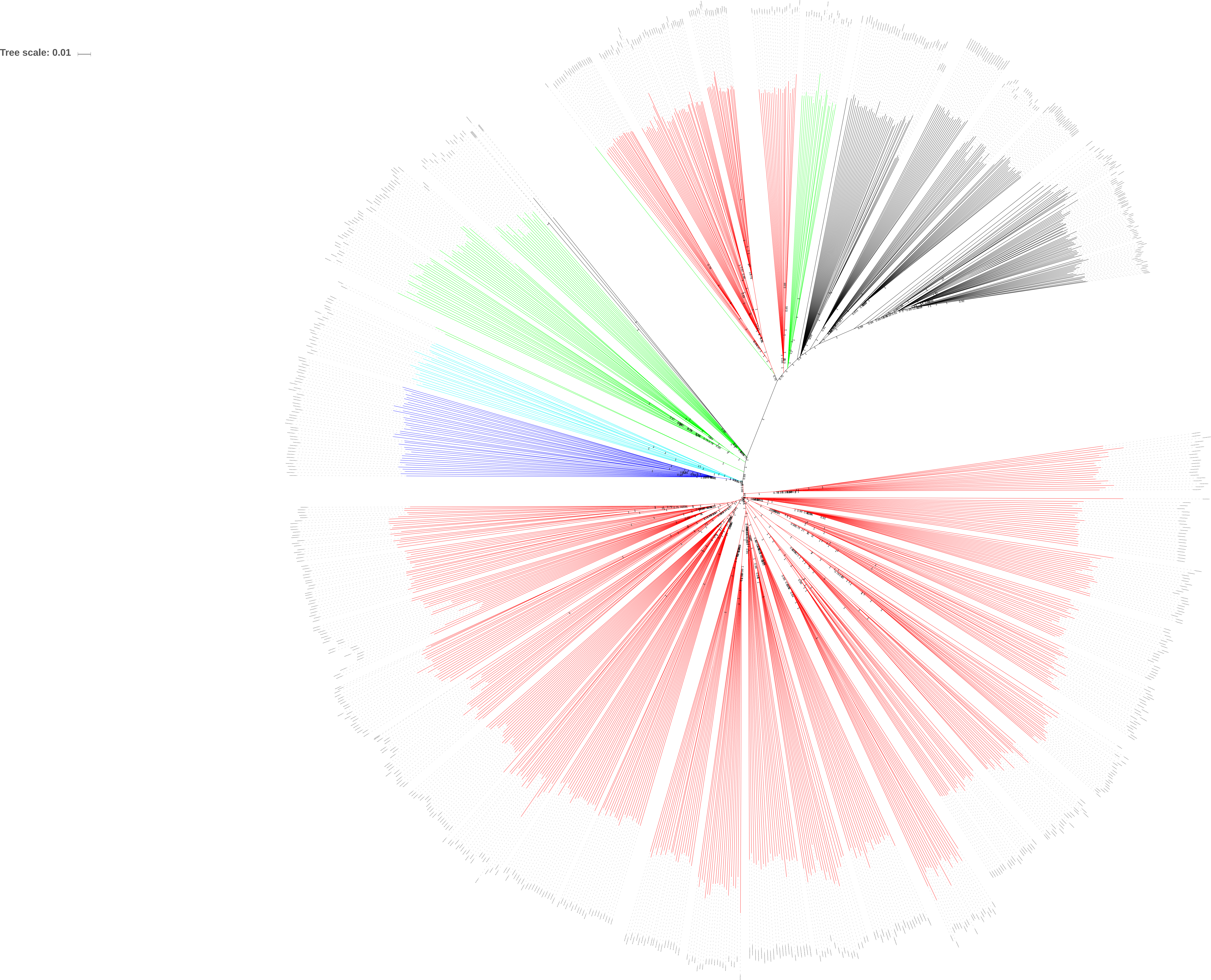
Tree scale: 0.01

Supplementary Figure 2. Ancestry models with ancestral populations (K=4, K=5, K=10, K=15, K=20, K=25, K=30, K=40). Breed names correspond to ABO: Abondance, AN: Angus, AUB: Aubrac, BALI: Bali, BAO: Baoule, BEFM: Beefmaster, BOR: Boran, BORG: Borgou, BR: Brahman, BRVH: Braunvieh, BSW: Brown Swiss, CANC: Canchim, CHA: French Charolais, CHCU: Cuban Charolais, GAS: Gascon, GEL: Gelbvieh, GIR: Gir, GNS: Guernsey, HFD: Hereford, HO: Holstein, JER: Jersey, KUR: Kuri, LAG: Lagune, LM: Limousin, MAAN: Maine-Anjou, MONT: Montbeliard, NDAM: N'Dama, NEL: Nelore, NORM: Normande, NRC: Norwegian Red, ONG: Ongole Grade, PIED: Piedmontese, PRP: French Red Pied Lowland, RMG: Romagnola, SAL: Salers, SGT: Santa Gertrudis, SIM: Simmental, SOM: Somba, TXLH: Texas Longhorn, VOS: Vosgienne, ZBO: Zebu Bororo, ZEB: East African Shorthorn Zebu, ZFU: Zebu Fulani, ZMA: Zebu from Madagascar.

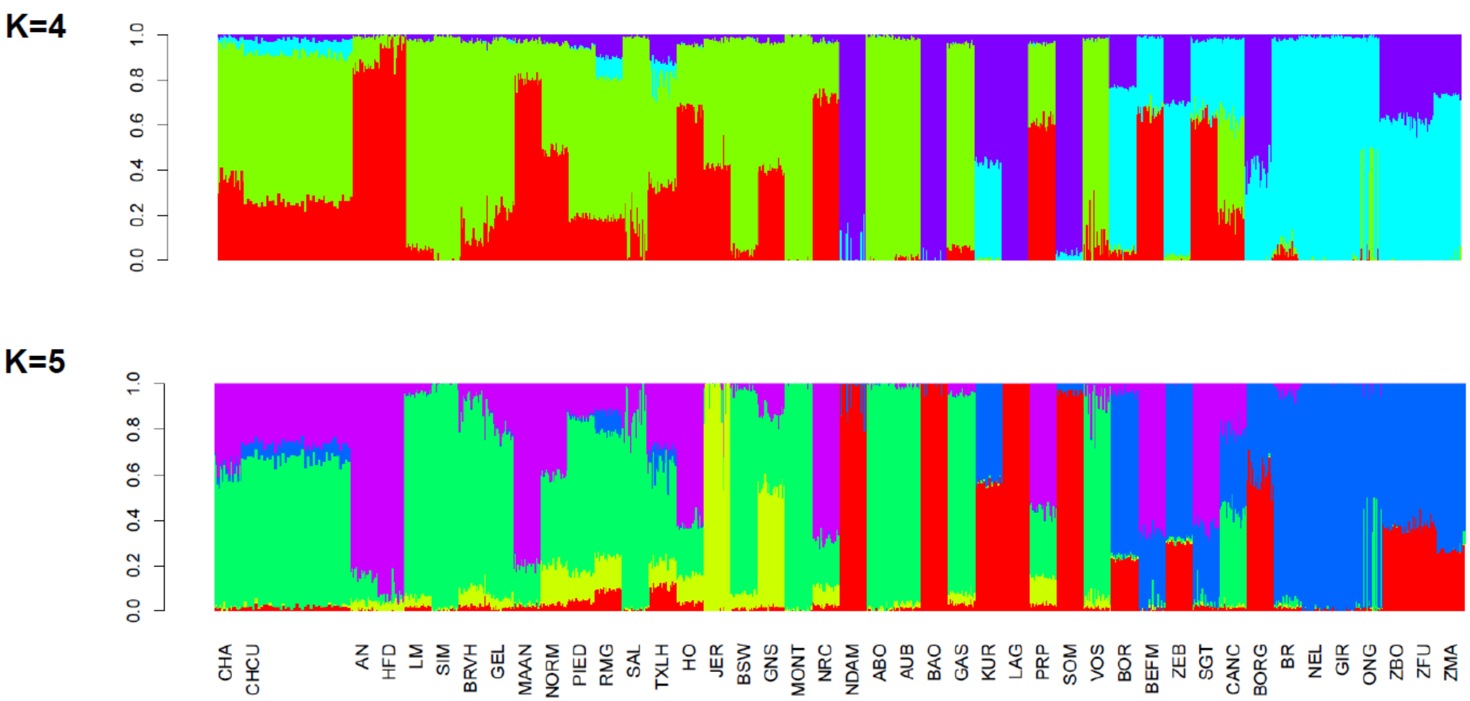

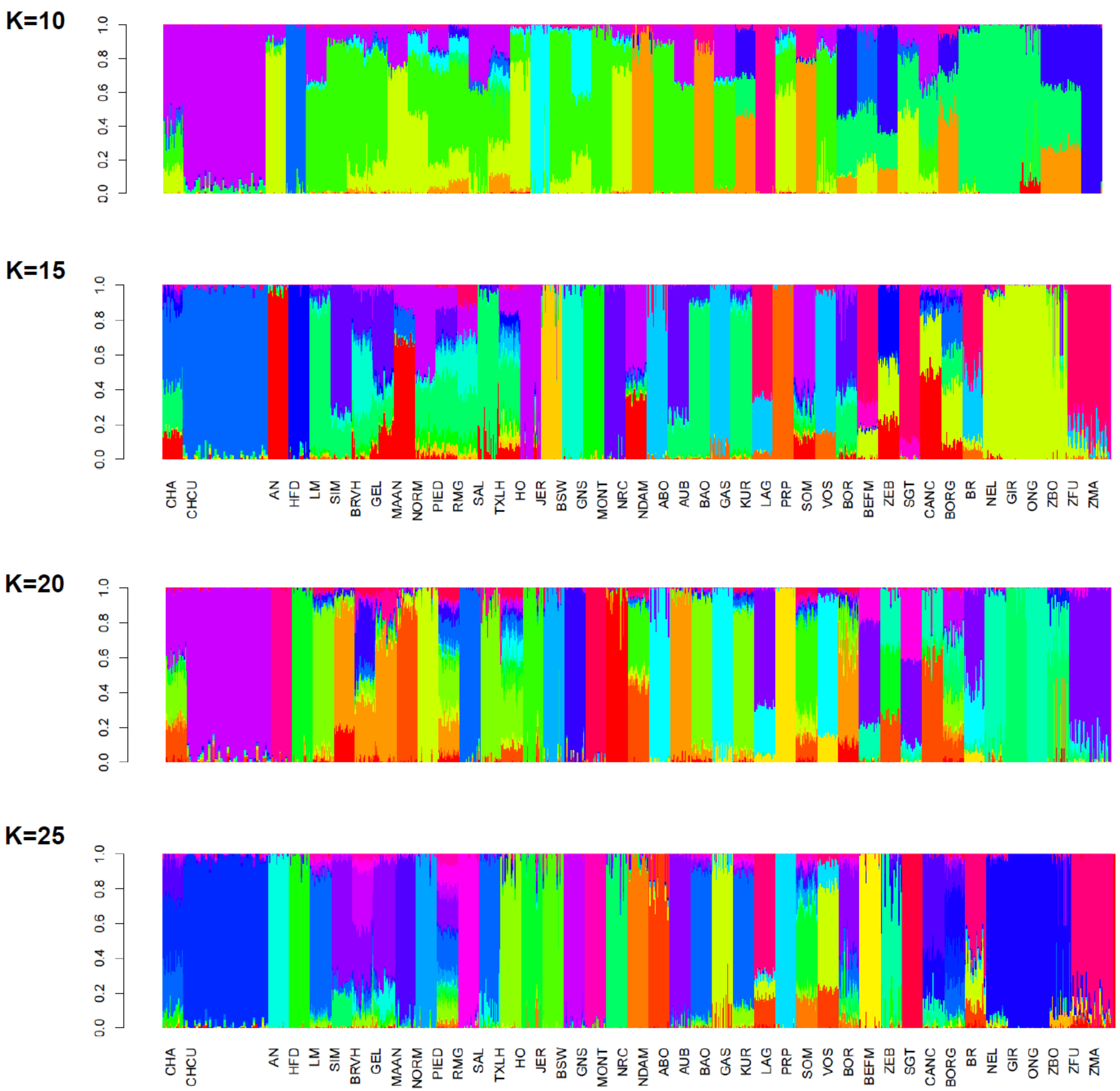
K=10
K=15
K=20
K=25
0.0 0.2 0.4 0.6 0.8 1.0
CHA CHCU AN HFD LM SIM BRVH GEL MAAN NORM PIED RMG SAL TXLH HO JER BSW GNS MONT NRC NDAM ABO AUB BAO GAS KUR LAG PRP SOM VOS BOR BEFM ZEB SGT CANC BORG BR NEL GIR ONG ZBO ZFU ZMA
CHA CHCU AN HFD LM SIM BRVH GEL MAAN NORM PIED RMG SAL TXLH HO JER BSW GNS MONT NRC NDAM ABO AUB BAO GAS KUR LAG PRP SOM VOS BOR BEFM ZEB SGT CANC BORG BR NEL GIR ONG ZBO ZFU ZMA

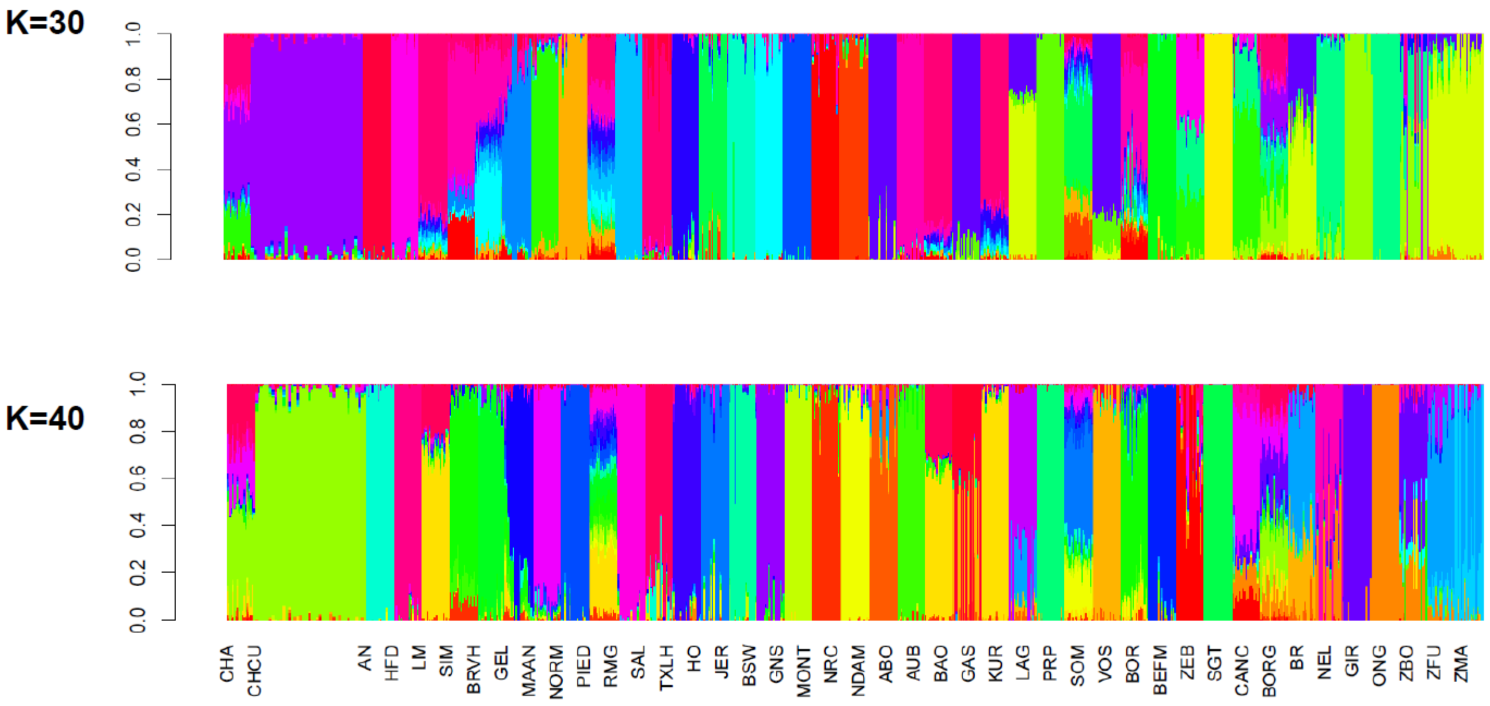

K=30
K=40
0.0
0.2
0.4
0.6
0.8
1.0
CHA
CHCU
AN
HFD
LM
SIM
BRVH
GEL
MAAN
NORM
PIED
RMG
SAL
TXLH
HO
JER
BSW
GNS
MONT
NRC
NDAM
ABO
AUB
BAO
GAS
KUR
LAG
PRP
SOM
VOS
BOR
BEFM
ZEB
SGT
CANC
BORG
BR
NEL
GIR
ONG
ZBO
ZFU
ZMA

BIBLIOGRAPHY

1. Ajmone-Marsan, P., Garcia, J. F., Lenstra, J. A. & and Globaldiv, C. On the Origin of Cattle: How Aurochs Became Cattle and Colonized the World. *Evolutionary Anthropology* **19,** 148–157 (2010).
2. Chen, S. *et al.* Zebu Cattle Are an Exclusive Legacy of the South Asia Neolithic. *Mol. Biol. Evol.* **27,** 1–6 (2010).
3. Loftus, R. T., MacHugh, D. E., Bradley, D. G., Sharp, P. M. & Cunningham, P. Evidence for two independent domestications of cattle. *Proc. Natl. Acad. Sci. U. S. A.* **91,** 2757–2761 (1994).
4. Magee, D. A., MacHugh, D. E. & Edwards, C. J. Interrogation of modern and ancient genomes reveals the complex domestic history of cattle. *Anim. Front.* **4,** 7–22 (2014).
5. Morales Padrón, F. *Historia del Descubrimiento y Conquista de América. Editorial GREDOS.* (1990).
6. López, D., Miriam, R., Díaz, J. & Menéndez, A. *Introducción y desarrollo del Charolais en Cuba. En: El Charolais cubano*. (Editorial Científico-Técnica, 1977).
7. L'Elevage, I. de. Résultats du contrôle des performances bovins allaitants. France, campagne 1990. *Collect. Résultats***,** 111 (1990).
8. Renand, G., Menendez-Buxadera, A., Krauss, D. & Menissier, F. Comparaison expérimentale du Charolais Cubain sélectionné en conditions tropicales avec du Charolais Français. *Renc. Rech. Ruminants* **4,** 230 (1997).
9. Ribas, M. Gene frecuencies in the blood group systems of the Cuban Charolais. *Ann. Génét. Sél. Anim.* **13,** 293–300 (1981).
10. Decker, E. J. *et al.* Worldwide Patterns of Ancestry, Divergence, and Admixture in Domesticated Cattle. *PLoS Genet* **10,** e1004254 (2014).
11. Gibbs, R. A. *et al.* Genome-wide survey of SNP variation uncovers the genetic structure of cattle breeds. *Science.* **324,** 528–532 (2009).
12. Porto, L. R. N. *et al.* Genomic divergence of zebu and taurine cattle identified through high-density SNP genotyping. *BMC Genomics* **14,** 876 (2013).
13. Weir, B. S. & Cockerham, C. C. Estimating F-Statistics for the Analysis of Population Structure. *Evolution (N. Y).* **38,** 1358 (1984).
14. Gautier, M., Laloe, D. & Moazami-Goudarzi, K. Insights into the Genetic History of French Cattle from Dense SNP Data on 47 Worldwide Breeds. *PLoS One* **5,** e13038 (2010).
15. Beghain, J. *et al.* Genome-wide linkage disequilibrium in the Blonde d'Aquitaine cattle breed. *J. Anim. Breed. Genet.* **130,** 294–302 (2013).
16. O'Brien, P. A. M. *et al.* Linkage disequilibrium levels in *Bos indicus* and *Bos taurus* cattle using medium and high density SNP chip data and different minor allele frequency distributions. *Livest. Sci.* **166,** 121–132 (2014).
17. Porto-neto, L. R., Kijas, J. W. & Reverter, A. The extent of linkage disequilibrium in beef cattle breeds using high-density SNP genotypes. *Genet. Sel. Evol.* **46,** 22 (2014).
18. Porto-Neto, L. R. *et al.* The genetic architecture of climatic adaptation of tropical cattle. *PLoS One* **9,** 11 (2014).
19. Howard, J. T. *et al.* Beef cattle body temperature during climatic stress: a genome-wide association study. *Int J Biometeorol* 1665–1672 (2014). doi:10.1007/s00484-013-0773-5
20. Hayes, B. J. *et al.* A Validated Genome Wide Association Study to Breed Cattle Adapted to an Environment Altered by Climate Change. *PLoS One* **4,** e6676 (2009).
21. Direito, I., Madeira, A., Brito, M. A. & Soveral, G. Aquaporin-5 : from structure to function and dysfunction in cancer. *Cell. Mol. Life Sci* **73,** 1623–1640 (2016).
22. Krane, C. M. *et al.* Salivary Acinar Cells from Aquaporin 5-deficient Mice Have Decreased

Membrane Water Permeability and Altered Cell Volume Regulation *. *J. Biol. Chem.* **276,** 23413–23420 (2001).
23. Sugimoto, N. *et al.* Upregulation of aquaporin expression in the salivary glands of heat-acclimated rats. *Sci. Rep.* **3,** 1763 (2013).
24. Mymrikov, E. V, Seit-nebi, A. S. & Gusev, N. B. Large potentials of small heat shock proteins. *Physiol Rev* **91,** 1123–1159 (2011).
25. Srikanth, K., Kwon, A., Lee, E. & Chung, H. Characterization of genes and pathways that respond to heat stress in Holstein calves through transcriptome analysis. *Cell Stress Chaperones* **22,** 29–42 (2017).
26. Kolli, V., Upadhyay, R. C. & Singh, D. Peripheral blood leukocytes transcriptomic signature highlights the altered metabolic pathways by heat stress in zebu cattle. *Res. Vet. Sci.* **96,** 102–110 (2014).
27. Kapila, N. *et al.* Impact of Heat Stress on Cellular and Transcriptional Adaptation of Mammary Epithelial Cells in Riverine Buffalo (*Bubalus Bubalis*). *PLoS One* **11,** e0157237 (2016).
28. Sun, M., Lee, C. J. & Shin, H. Reduced nicotinic receptor function in sympathetic ganglia is responsible for the hypothermia in the acetylcholinesterase knockout mouse. *J Physiol* **578,** 751–764 (2007).
29. Cheng, C. *et al.* Functional genomics study of acute heat stress response in the small yellow follicles of layer-type chickens. *Sci. Rep.* **8,** 1320 (2018).
30. Mailloux, R. J. & Harper, M. Uncoupling proteins and the control of mitochondrial reactive oxygen species production. *Free Radic. Biol. Med.* **51,** 1106–1115 (2011).
31. Argyropoulos, G. & Harper, M.-E. highlighted topics. *J Appl Physiol 92* **92,** 2187–2198 (2002).
32. Vidal-puig, A. J. *et al.* Energy Metabolism in Uncoupling Protein 3 Gene Knockout Mice. *J. Biol. Chem.* **275,** 16258–16266 (2000).
33. Sun, H. *et al.* Transcriptome responses to heat stress in hypothalamus of a meat-type chicken. *J. Anim. Sci. Biotechnol.* **6,** 6 (2015).
34. Salgado, R. M. *et al.* Mitochondrial efficiency and exercise economy following heat stress : a potential role of uncoupling protein 3. *Physiol. Rep.* **5,** e13054 (2017).
35. Ramey, R. H., Decker, E. J., McKay., S. D., Schnabel, D. R. & Taylor, F. J. Detection of selective sweeps in cattle using genome-wide SNP data. *BMC Genomics* **14,** (2013).
36. Gutiérrez-Gil, B., Arranz, J. J. & Wiener, P. An interpretive review of selective sweep studies in Bos taurus cattle populations: Identification of unique and shared selection signals across breeds. *Front. Genet.* **6,** (2015).
37. Bahbahani, H. *et al.* Signatures of positive selection in African Butana and Kenana dairy zebu cattle. *PLoS One* **13,** e0190446 (2018).
38. Niskanen, A. *et al.* MHC variability supports dog domestication from a large number of wolves: high diversity in Asia. *Heredity (Edinb).* **110,** 80–85 (2013).
39. Groot, N. G. De *et al.* Evidence for an ancient selective sweep in the MHC class I gene repertoire of chimpanzees. *PNAS* **99,** 11748–11753 (2002).
40. Meyer, D. & Thomson, G. How selection shapes variation of the human major histocompatibility complex: a review. *Ann. Hum. Genet.* **65,** 1–26 (2001).
41. Blum, J. S., Wearsch, P. A. & Cresswell, P. Pathways of Antigen Processing. *Annu Rev Immunol* **31,** 443–473 (2013).
42. Rikihisa, Y. Mechanisms of Obligatory Intracellular Infection with Anaplasma phagocytophilum. *Clin. Microbiol. Rev.* **24,** 469–489 (2011).
43. Homer, M. J., Aguilar-delfin, I., S A M R, T. I., Krause, P. J. & Persing, D. H. Babesiosis. *Clin. Microbiol. Rev.* **13,** 451–469 (2000).
44. Marín, A. L. M., Hernández, M. P., Alba, L. P., Castro, G. G. & Pardo, D. C. Metabolismo

de los lípidos en los rumiantes - Lipid metabolism in ruminants. *REDVET Rev. electrón. vet.* **11,** 1–21 (2010).
45. Ramayo-Caldas. Yuliaxis, Renand, G., Ballester, M., Saintilan, R. & Rocha, D. Multi - breed and multi - trait co - association analysis of meat tenderness and other meat quality traits in three French beef cattle breeds. *Genet. Sel. Evol.* **48,** 1–9 (2016).
46. Huang, W. *et al.* Global transcriptome analysis identifies differentially expressed genes related to lipid metabolism in Wagyu and Holstein cattle. *Front. Immunol.* **7,** 5278 (2017).
47. Ramayo-Caldas, Y., Fortes, M. R. S., Hudson, N. J. & Bolormaa, S. A marker-derived gene network reveals the regulatory role of PPARGC1A , HNF4G , and FOXP3 in intramuscular fat deposition of beef cattle 1. *J. Anim. Sci.* **92,** 2832–2845 (2014).
48. Sánchez, M. P. Índices de malnutrición proteica y proteico-energética en musculo gastrocnemio de ratas por déficit dietario de fenilalanina, tirosina y triptófano. (INSTITUTO DE NUTRICION, 1988).
49. McCormack, W. P. *et al.* Oral nutritional supplement fortified with beta-alanine improves physical working capacity in older adults: A randomized, placebo-controlled study. *Exp. Gerontol.* **48,** 933–939 (2013).
50. Purcell, S. *et al.* PLINK: a toolset for whole-genome association and population-based linkage analysis. *Am. J. Hum. Genet.* **81,** 559–575 (2007).
51. Price, L. A. *et al.* Principal components analysis corrects for stratification in genome-wide association studies. *Nat. Genet.* **38,** 904–909 (2006).
52. Kumar, S., Stecher, G. & Tamura, K. MEGA7: Molecular Evolutionary Genetics Analysis Version 7 . 0 for Bigger Datasets. *Mol. Biol. Evol.* **33,** 1870–1874 (2016).
53. Saitou, N. & Nei, M. The Neighbor-joining Method: A New Method for Reconstructing Phylogenetic Trees '. *Mol. Biol. Evol.* **4,** 406–425 (1987).
54. Nei, M. & Kumar, S. *Molecular Evolution and Phylogenetics. Oxford University Press, New York.* (2000).
55. Alexander, D. H., Novembre, J. & Lange, K. Fast model-based estimation of ancestry in unrelated individuals. *Genome Res.* **19,** 1655–1664 (2009).
56. Tang, K., Thornton, R. K. & Stoneking, M. A New Approach for Using Genome Scans to Detect Recent Positive Selection in the Human Genome. *PLoS Biol.* **5,** e171 (2007).
57. Benjamini, Y. & Hochberg, Y. Controlling the False Discovery Rate: A Practical and Powerful Approach to Multiple Testing. *J. R. Stat. Soc. Ser. B* **57,** 289–300 (1995).
58. Qanbari, S. *et al.* A genome-wide scan for signatures of recent selection in Holstein cattle. *Anim. Genet.* **41,** 377–389 (2010).
59. Larkin, D. M. *et al.* Whole-genome resequencing of two elite sires for the detection of haplotypes under selection in dairy cattle. *PNAS* **109,** 7693–7698 (2012).
60. Druet, T., Pérez-Pardal, L., Charlier, C. & Gautier, M. Identification of large selective sweeps associated with major genes in cattle. *Anim. Genet.* **44,** 758–762 (2013).
61. Kim, E. *et al.* Effect of Artificial Selection on Runs of Homozygosity in U.S. Holstein Cattle. *PLoS One* **8,** 1–14 (2013).
62. Zhao, F., Mcparland, S., Kearney, F., Du, L. & Berry, D. P. Detection of selection signatures in dairy and beef cattle using high-density genomic information. *Genet. Sel. Evol.* **47,** 1–12 (2015).
63. Rothammer., S., Seichter., D., Förster., M. & Medugorac., I. A genome-wide scan for signatures of differential artificial selection in ten cattle breeds. *BMC Genomics* **14,** (2013).
64. Flori, L. *et al.* The Genome Response to Artificial Selection: A Case Study in Dairy Cattle. *PLoS One* **4,** e6595 (2009).
65. Hosokawa, D. *et al.* Identification of divergently selected regions between Japanese Black and Holstein cattle using bovine 50k SNP array. *Anim. Sci. J.* **83,** 7–13 (2012).
66. Bahbahani, H. *et al.* Signatures of Selection for Environmental Adaptation and Zebu ×

Taurine Hybrid Fitness in East African Shorthorn Zebu. *Front. Genet.* **8,** (2017).
67. Utsunomiya, Y. T. *et al.* Genome-wide association study for birth weight in Nellore cattle points to previously described orthologous genes affecting human and bovine height. *RESEARCH* **14,** (2013).
68. Xu, L. *et al.* Genomic Signatures Reveal New Evidences for Selection of Important Traits in Domestic Cattle. *Mol. Biol. Evol.* **32,** 711–725 (2014).
69. Kemper, K. E., Saxton, S. J., Bolormaa, S., Hayes, B. J. & Goddard, M. E. Selection for complex traits leaves little or no classic signatures of selection. *BMC Genomics* **15,** (2014).
70. Glick, G. *et al.* Signatures of contemporary selection in the Israeli Holstein dairy cattle. *Anim. Genet.* **43,** 45–55 (2012).
71. Gurgul, A. *et al.* Genome-wide characteristics of copy number variation in Polish Holstein and Polish Red cattle using SNP genotyping assay. *Genetica* **143,** 145–155 (2015).
72. Lee, H. *et al.* Deciphering the Genetic Blueprint behind Holstein Milk Proteins and Production. *Genome Biol. Evol.* **6,** 1366–1374 (2014).
73. Bomba, L. *et al.* Relative extended haplotype homozygosity signals across breeds reveal dairy and beef specific signatures of selection. *Genet. Sel. Evol.* **47,** (2015).
74. Qanbari, S. *et al.* Classic Selective Sweeps Revealed by Massive Sequencing in Cattle. *PLoS Genet* **10,** e1004148 (2014).
75. O'Brien, A. M. P. *et al.* Assessing signatures of selection through variation in linkage disequilibrium between taurine and indicine cattle. *Genet. Sel. Evol.* **46,** (2014).
76. Qanbari, S. *et al.* Application of site and haplotype-frequency based approaches for detecting selection signatures in cattle. *BMC Genomics* **12,** (2011).
77. Somavilla, A. *et al.* A genome-wide scan for selection signatures in Nellore cattle. *Anim Genet.* **45,** 771–781 (2014).
78. Fan, H. *et al.* Genome-wide detection of selective signatures in Simmental cattle. *J. Appl. Genet.* **55,** 343–351 (2014).
79. Chan, E. K. F., Nagaraj, S. H. & Reverter, A. The evolution of tropical adaptation: comparing taurine and zebu cattle. *Anim. Genet.* **41,** 467–477 (2010).
80. MacEachern, S., Hayes, B., Mcewan, J. & Goddard, M. An examination of positive selection and changing effective population size in Angus and Holstein cattle populations (Bos taurus) using a high density SNP genotyping platform and the contribution of ancient polymorphism to genomic diversity in domestic ca. *BMC Genomics* **10,** (2009).
